# Neuroprotective efficacy of berberine and caffeine against rotenone‐induced neuroinflammatory and oxidative disturbances associated with Parkinson’s disease via inhibiting α-synuclein aggregation and boosting dopamine release

**DOI:** 10.1007/s10787-025-01661-w

**Published:** 2025-03-09

**Authors:** Tasnim S. Waheeb, Mohammad A. Abdulkader, Doaa A. Ghareeb, Mohamed E. Moustafa

**Affiliations:** 1 Department of Biochemistry, Faculty of Science, Alexandria University, Alexandria, 21511 Egypt; 2 Bio-Screening and Preclinical Trial Lab, Biochemistry Department, Faculty of Science, Alexandria University, Alexandria, Egypt; 3 Center of Excellence for Drug Preclinical Studies (CE-DPS), Pharmaceutical and Fermentation Industry Development Center, City of Scientific Research and Technological Applications (SRTA-City), New Borg El Arab, Alexandria Egypt; 4 Research Projects Unit, Pharos University in Alexandria, Canal El Mahmoudia Street, Beside Green Plaza Complex, Alexandria, 21648 Egypt

**Keywords:** Natural combination therapy, Motor impairment, Neurodegenerative diseases, Rotenone model, Protein phosphatase 2A, Tyrosine hydroxylase, Molecular docking

## Abstract

Parkinson's disease (PD) is characterized by motor impairment, glial-mediated inflammation, redox imbalance, and α-synuclein (α-syn) aggregation. Conventional therapies relieve early PD symptoms, but they do not repair dopaminergic neurons. Berberine (BBR) and caffeine (CAF), both natural alkaloids, exhibited neuroprotective effects in many neurodegenerative disorders. Consequently, we hypothesized that the combination of BBR and CAF therapies would offer protection against PD-related impairments in the rotenone (ROT)-induced rat model when compared to the commercial drug, metformin (MTF). Our results showed that the combined administration of BBR (25 mg/kg/day) and CAF (2.5 mg/kg/day) for four weeks prevented motor deficits, weight reduction, dopamine (DA) depletion, and monoamine oxidase (MAO) activity in ROT-induced rats in comparison with monotherapy of BBR and CAF along with MTF. This combination produced a notable neuroprotective effect by reducing tumor necrosis factor (TNF)-α and interleukin-16 (IL-6) in midbrain of rats. BBR and CAF combinations markedly normalized tyrosine hydroxylase (TH) levels and decreased total α-syn and α-syn-p^ser129^ aggregation and increased protein phosphatase 2A (PP2A) levels. Histological analysis indicated that damaged neurons exhibited significant amelioration with the co-administration of BBR and CAF. The molecular docking results indicated that both BBR and CAF had notable binding affinity for the protein pocket surrounding the α-syn, PP2A, and TH in comparison to MTF. They are predicted to serve as effective inhibitors of enzyme-mediated phosphorylation of α-syn-^pser129^. Conclusively, combined BBR and CAF administration presents a novel strategy for neuroprotection by blocking the initial events in PD incidence, demonstrating considerable anti-oxidative and anti-inflammatory benefits relative to MTF.

**Graphical abstract:**

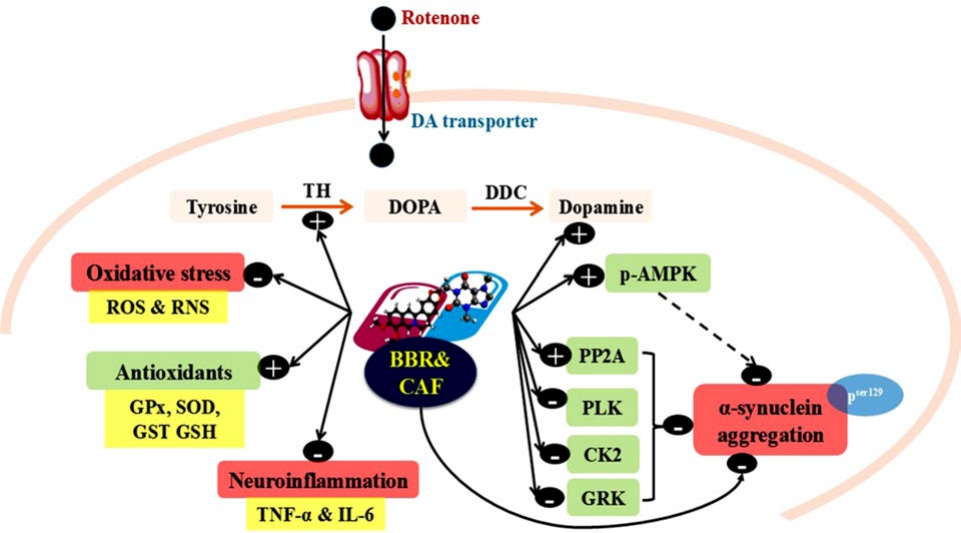

**Electronic supplementary material:**

The online version of this article (10.1007/s10787-025-01661-w) contains supplementary material, which is available to authorized users.

## Introduction

Approximately 1–3% of the world's population over the age of fifty suffer from Parkinson’s disease (PD). It is considered as the second most prevalent form of dementia, following Alzheimer's disease. Parkinson's disease is more prevalent in high-income countries; however, it is also emerging as a significant health issue in low-income nations (Pereira et al. 2024). The initiation and progression of PD have been associated with alterations in biochemical pathways such as mitochondrial impairment, production of inflammatory cytokines, impaired protein homeostasis resulting from defects in the ubiquitin–proteasome system, and a gradual loss of dopaminergic neurons that causes a lack of dopamine (DA) in the striatum (Jin et al. 2024). A model of PD in rodents can be induced by subcutaneous administration of rotenone (ROT), a chemical pesticide capable of blocking electron transport chain complex I in mitochondria (Ibarra-Gutiérrez et al. 2023). It has been shown that ROT can selectively target dopaminergic cells both in vivo and in vitro (Lawana and Cannon 2020). Rotenone is a lipophilic compound that can cross the blood–brain barrier and cell membranes of dopaminergic neurons to generate α-synuclein (α-syn) fibrils, leading to the induction of PD in rodents (Giraldo-Berrio et al. 2024).

The predominant post-translational alteration of α-syn is the hyperphosphorylation of the serine residue at position 129, resulting in phospho-Ser129-α-syn (Canerina-Amaro et al. 2019). This process is facilitated by casein kinases (CK2), G protein-coupled receptor kinases (GKR), and Polo-like kinases (PLK) (Kawahata et al. 2022). The resultant α-syn aggregation contributes to the pathogenesis of PD by elevated reactive oxygen species (ROS) generation (Sohrabi et al. 2023) and inflammatory cytokines including tumor necrosis factor-alpha (TNF-α) and interleukin-1 (IL-1) in the dopaminergic neurons (Cardinale et al. 2021). α-syn reduces DA synthesis via reducing tyrosine hydroxylase (TH) activity via protein phosphatase 2A (PP2A) methylation and TH dephosphorylation at Ser 40 (Hua et al. 2015). Further, the overexpression of α-syn directly increases monoamine oxidase (MAO) activity, which catalyzes the oxidative deamination of monoamine neurotransmitters such as DA production and increases ROS generation (Wu et al. 2021). In PD, DA depletion causes resting tremors, slowing movement, stiffness anomalies, and involuntary movements (Ramesh and Arachchige 2023). Over and above, PP2A plays a significant role during PD progression. It contains a highly conserved dimeric core of catalytic (C), scaffold-like (A), and regulatory (B) subunits that direct it to distinct substrate phosphoproteins. The binding selectivity of PP2A is modulated by carboxy methylation of its C subunit at leucine-309, which enhances the binding affinity of AC dimer with specific regulatory B subunits. It was reported that the brains of dementia patients had considerably decreased phosphatase activity and PP2A carboxyl methylation levels compared to healthy controls (Park et al. 2016).

According to WHO data, 80% of underdeveloped nations depend on alternative treatments. Herbal therapies and combinations of phytocompounds with various pharmacological and biological applications have gained national, economic, and scientific importance. Phytochemicals such as flavonoids, polyphenols, tannins, alkaloids, and sterols have antioxidant, anticholinesterase, anti-inflammatory, and anti-amyloidogenic properties, which improve neuronal function (Koul et al. 2023). Berberine (BBR) is an isoquinoline alkaloid extracted from various plants and traditionally used in Chinese medical practice. Recent studies have demonstrated the diverse pharmacological activities of BBR, such as anti-inflammatory, antiviral, anti-tumor, antibacterial, antifungal, anti-diarrheal, anti-diabetic, and anti-dyslipidemic pharmacological effects (Imenshahidi and Hosseinzadeh 2019; Wang et al. 2021; Khoshandam et al. 2022; Gasmi et al. 2024). Berberine can pass through the blood–brain barrier and inhibits MAO A and B that are localized in the outer membrane of mitochondria in dopaminergic neurons leading to a decline in DA level (Fan et al. 2019).

Additionally, caffeine (CAF) is a xanthine alkaloid derived from tea (*Camellia sinensis*), the leaves and seeds of coffee (*Coffea arabica*), and chocolate (*Theobroma cacao* L.). It can easily cross the blood–brain barrier due to its high lipophilicity (Song et al. 2024). The recent literature on CAF showed its neuroprotective benefits by inhibiting MAO-B and increasing DA levels, improving motor symptoms (Boulaamane et al. 2022). CAF may activate protein phosphatase 2A and inhibit α-syn aggregation (Yan et al. 2018). Also, recent research found that CAF reduces astrocyte and microglial activation, reducing inflammatory processes and lipid peroxidation while lowering ROS (Hosny et al. 2019).

Given the molecular connection between PD and type 2 diabetes, both conditions may benefit from similar treatments (Bantounou et al. 2024). In addition, previous animal studies indicate that anti-diabetic medications may be a potential neuroprotective therapy (Chen et al. 2021). In particular, metformin (MTF) is the first-line drug for treating insulin-resistant type 2 diabetes mellitus (Panfoli et al. 2021). Metformin can rapidly cross the blood–brain barrier and defend against dementia (Cui et al. 2024). It enhances PP2A expression and reduces α-syn phosphorylation and aggregation, inflammation, and oxidative stress associated with PD development (Ping et al. 2020). It can also enhance DA turnover and decrease the loss of TH protein (Paudel et al. 2020). Regrettably, long-term high-dose MTF treatment may raise PD risk due to vitamin B12 insufficiency and gastrointestinal tract irritation, including bloating, flatus, nausea, diarrhea, and constipation. Vitamin B12 directly binds to α-syn protein, thereby decreasing fibrillation and cytotoxicity. Long-term use of MTF may accelerate the pathogenesis of Parkinson's disease by promoting α-syn aggregation, even though it may prevent its propagation (Alrouji et al. 2024).

Unfortunately, there are significant negative effects linked to the current medications used to treat PD. Therefore, it is necessary to investigate innovative combination therapies that employ natural herbal active compounds, which may avoid existing limitations and exhibit neuroprotective effects through synergistic or cumulative interactions by targeting multiple cellular pathways. In light of the above-mentioned rationale, the main aim of this research was to investigate the overall neuroprotective effects of combined BBR and/or CAF as a new approach compared to MTF treatment on neurochemical deficits induced by ROT in rats. This study examines the mechanisms through which these compounds influence oxidative stress-mediated neuroinflammation. We examined the alterations in DA, MAO, TH, total α-Syn, α-Syn-p^ser129^, and PP2A levels in the midbrains of the rats treated with ROT. In addition, this study investigated the molecular docking of BBR, CAF, and MTF with specific protein targets involved in PD pathogenesis.

## Material and methods

### Animals

Sixty male Sprague–Dawley rats ranging between 200 and 220 g were acquired from the Alexandria Graduate Studies and Research Institute, Alexandria University, Egypt. Five rats were housed in each box and animals were given free water and a diet. Rats were kept in a precise setting of a 12-h light–dark cycle, 20–25 ℃ temperature, and 35–70% humidity. All animal procedures followed Alexandria University's Medical School Ethics Committee's (approval number AU 04191123101) and NIH guidelines on the management and utilization of laboratory animals.

### Drug preparation

Rotenone (ab143145) was purchased from Abcam (Cambridge, UK). It was suspended in sunflower oil at a concentration of 1.5 mg/mL before use. BBR chloride, MTF hydrochloride, and CAF were purchased from Sigma-Aldrich (St. Louis, MO, USA). BBR and CAF were dissolved in a 20% polyethylene glycol solution, while MTF hydrochloride was dissolved in distilled water.

### Animal treatment

**Fig. 1:**
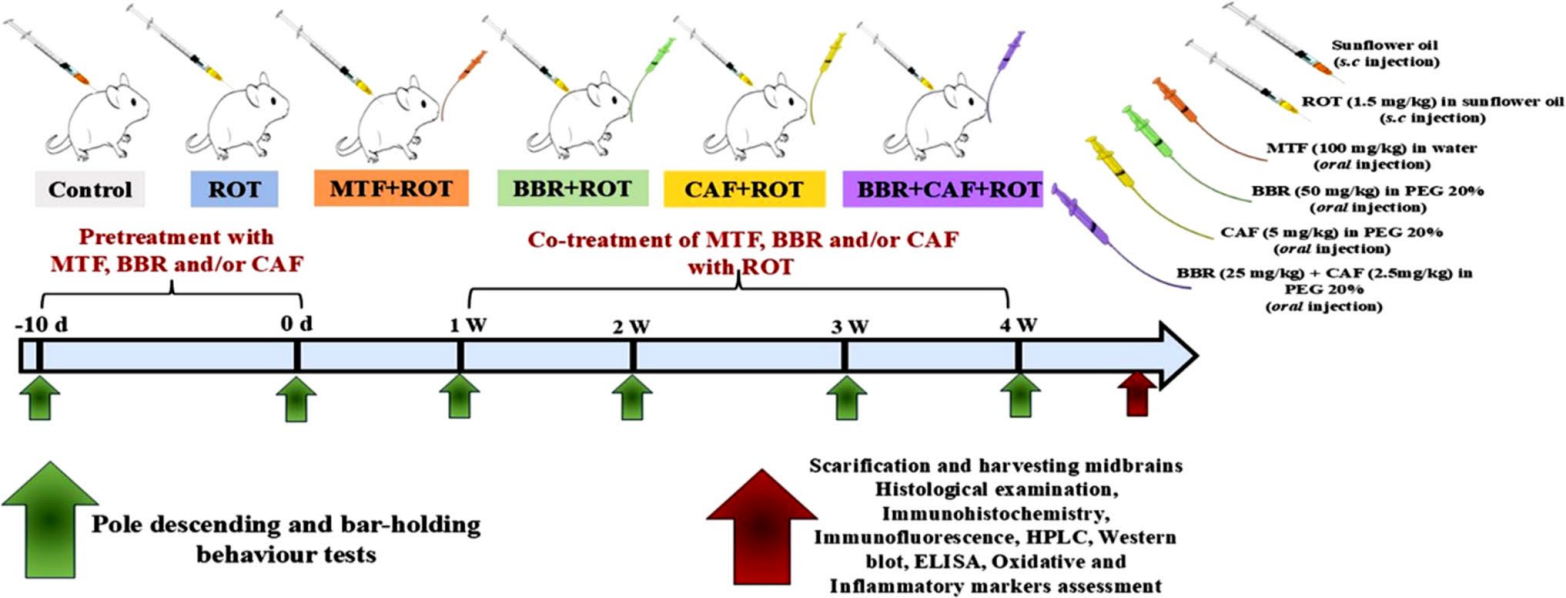
A schematic diagram describing the study design. *ROT* rotenone, *MTF* metformin, *BBR* berberine, *CAF* caffeine

After the adaptation of animals for a week, rats were randomly allocated into six groups (10 rats/group) as follows: group 1 (Control): rats were administered (0.2 mL) sunflower oil subcutaneously (s.c). Group 2 (ROT-induced): rats received 1.5 mg/kg ROT in sunflower oil subcutaneously five times a week for four weeks (Zhang et al. 2017). Group 3: (MTF + ROT) rats received 100 mg/kg MTF via oral gavage for 10 days before ROT administration, followed by daily treatment with MTF and ROT five times a week for 4 weeks (Soraya et al. 2012). Group 4: (BBR + ROT) rats orally received 50 mg/kg BBR daily for ten days before ROT administration, followed by daily treatment with BBR and ROT five times a week for 4 weeks (Ghareeb et al. 2015). Group 5: (CAF + ROT) rats orally received 5 mg/kg CAF daily for ten days before ROT administration, followed by daily treatment with CAF and ROT five times a week for 4 weeks (Davoodi et.al 2014). Group 6: (BBR + CAF + ROT) rats orally received 2.5 mg/kg and 25 mg/kg of CAF and BBR, respectively, via oral gavage and co-treated with ROT 5 times a week for four weeks (Fig. 1). All rats were sacrificed after isoflurane anesthesia one day after the last treatment, the serum was separated from blood samples, and brain tissues were harvested and kept at − 80 ℃ until analysis. Midbrain tissue was harvested, washed in ice-cold PBS buffer solution of pH 7.4, and divided into two sections. The first section was used for the determination of biochemical and molecular markers. The second half of the brain was maintained in a 10% formalin solution for 24 h, rinsed with water and repeated ethanol dilutions, and used for histological examination, immunohistochemistry, and immunofluorescent labeling of tyrosine hydroxylase and total α-syn.

### Pole-based motor and bar holding behavior assessments

The pole-based motor assessment measured movement defects associated with the basal ganglia. In this test, rats were positioned on the edge of a vertical wooden pole that was 70 cm tall and 2 cm in diameter. The base of this pole was placed within a cage containing bedding material. When placed on the top of the pole, the rodent orients itself downward and descends its length. First, rats were trained for two days, then rats from each group in this study were given five trials every week and the best time of descending to the pole's base was recorded (Matsuura et al. 1997). The horizontal holding bar test was used to examine the strength and coordination of the rodents' forelimbs. The rats were held by the tail and aligned perpendicular to the bar. They were raised and allowed to grasp the horizontal bar's center with only their forepaws, after that their tails were released and the stopwatch began. Each group of rodents got multiple weekly trials, and their averages were recorded (Deacon 2013). 

### Assessment of the total amount of protein

The brain tissue was homogenized in the ice-cold buffer of 100 mM Tris, pH 7.4, 1 mM EGTA, 150 mM NaCl and EDTA, 0.5% sodium deoxycholate, and 1% Triton X-100 containing protease inhibitor (Roche, Mannheim, Germany). The homogenate underwent centrifugation at 12,000 rpm for 20 min at 4 ℃. The protein concentration was evaluated with the technique established by previous method (Lowry et al. 1951).

### Determination of dopamine by high-performance liquid chromatography

A weight of 1 mg of the midbrain was homogenized in 100 µL of 0.2 M perchloric acid centrifuged at 12,000 rpm at 4 ℃ for 20 and subsequently filtered through a disposable syringe filter (PTFE membrane filters, pore size 0.22 mm, Advantec MFS, Tokyo, Japan). The standard stock concentration was 40 µg/mL of DA hydrochloride (H8502-25G, Sigma-Aldrich). Waters 2690 Alliance HPLC system with a Waters 996 photodiode array detector (Spectra Lab Scientific Inc, Canada) was used to determine DA levels at 254 nm. The mobile phase was 0.1% phosphoric acid in water and 99:1 acetonitrile (Gu et al. 2016).

### Monoamine oxide assay

Midbrain tissue was homogenized in 50 mM potassium phosphate, pH of 7.5, and centrifuged at 4000 rpm for 15 min at 4 ℃; afterward, the supernatant was collected. Brain homogenate was mixed with 500 mM p-tyramine substrate, and potassium phosphate buffer pH 7.6. The absorbance was assessed at 250 nm absorbance after 30 and 90 s (Sandler et al. 1981). 

### Immunohistochemistry for α-synuclein and tyrosine hydroxylase

After 24 h in 10% formalin buffered solution (pH 7.4), tissue was washed with saline and fixed at 56 ℃ in a hot air oven before paraffinization. Five-mm-thick midbrain slices were deparaffinized from paraffin blocks in xylene and immersed in deionized water with 0.01% hydrogen peroxide for 10 min to diminish intrinsic peroxidase activity. Tissue slices were treated overnight at 4 ℃ with rabbit polyclonal anti-α-syn (1:1000, Thermo Fisher Scientific, Massachusetts, USA) and rabbit monoclonal anti-TH (1:200, Novus Biologicals, Colorado, US) antibodies. Tissue sections were incubated with avidin–biotin complex (ABC) reagent (#PK-6104, Vectastain ABC-HRP Kit, Vector Laboratories Inc, CA, USA) for one hour and were tagged with horseradish peroxidase (#F9291) coupled with colored diaminobenzidine substrate (DAB) Sigma-Aldrich (St. Louis, MO, USA) for 6–10 min to identify antigen–antibody complexes (Kim et al. 2016). Stained sections were examined by light microscope and photographed by digital camera (Nikon Corporation Co., Ltd., Japan). ImageJ (Version 1.53) was used to measure the intensities of images.

### Immunofluorescence analysis of α-Syn-p^ser129^

Midbrain sections of 5 mm thick were dewaxed with xylene and rehydrated with ethanol and distilled water. Each section was fixed with 10% formaldehyde. A 0.1% Triton X-100 was added followed by 1% BSA in PBS for blocking. The incubation with α-Syn-p^ser129^ primary monoclonal antibody (PA1-4686 1:2000) was carried out overnight at 4 ℃. Alexa Fluor 488 (A20181 Invitrogen, USA, 1:2000) secondary antibodies were added to the sections and incubation was carried out for 60 min. The sections were stained with 4′,6-diamidino-2-phenylindole (DAPI) and then mounted with Fluoromount-G (Southern Biotechnology, USA) (Im et al. 2019). The visualization was carried out using confocal laser scanning microscopy (LEICA, DMi8, Mannheim/Wetzlar, Germany).

### Western blotting for protein phosphatase 2A

RIPA lysis buffer with a phosphatase/protease inhibitor cocktail (Roche, Mannheim, Germany) was used to extract the cellular proteins. A weight of 5 mg of midbrain tissue was homogenized with 300 μL of ice-cold lysate buffer and centrifuged at 12,000 rpm for 20 min at 4 ℃. An aliquot of the supernatant was used to quantify proteins (Lowry et al. 1951). An aliquot of 20 μg of proteins was loaded into each well of 12% SDS gel, and the gel was run at 90 V for 1 h. Proteins were blotted onto nitrocellulose membranes (#1620094, Bio-Rad, Hercules, CA). Membranes were blocked with 1X Tris-buffered saline/0.1% Tween 20 (TBST) and 5% BSA for 1 h at room temperature. Primary antibodies specific for protein phosphatase 2A (PP2A) (PA5-17510, Invitrogen, Thermo Fisher Scientific, USA, dilution 1:1000) or β-actin (#4970S Cell Signaling Technology, Danvers, MA, USA, dilution 1:1000) were added for overnight at 4 ℃. The nitrocellulose membrane was incubated at room temperature for 2 h with a goat anti-rabbit alkaline phosphatase-conjugated secondary antibody (#7054, Cell Signaling Technology, Danvers, MA, USA dilution 1:1000). NBT/BCIP substrate (#34042, Invitrogen, Thermo Fisher Scientific, USA) revealed protein bands. ImageJ (version 1.53) was used to measure band intensities.

### Determination of neuroinflammatory mediators and p-AMPK levels by ELISA

The midbrain tissue was homogenized in the ice-cold buffer of 100 mM Tris, pH 7.4, 1 mM EGTA, 150 mM NaCl and EDTA, 0.5% sodium deoxycholate, and 1% Triton X-100 containing protease inhibitor (Roche, Mannheim, Germany). The homogenate underwent centrifugation at 12,000 rpm for 20 min at 4 ℃. The ELISA kits for determination of rat TNF-α (EK0526) (Boster, Pleasanton, CA, USA), IL-6 (CSB-E04640r) (Cusabio, Houston, TX, USA), and p-AMPK (MBS7230575) (MyBioSource, San Diego, CA, USA) were used according to the manufacturer's instructions.

### Assessments of oxidative and antioxidant stress markers

The malondialdehyde (MDA) concentrations in midbrain tissue homogenate were assessed utilizing the thiobarbituric acid reactive substances (TBARS) method. The methodology is based on the variation in absorbance of pink chromogen at 532 nm, and MDA values were reported as nmol/mg protein (Kei 1978). A previous standard protocol was followed to assess nitric oxide (NO) and xanthine oxidase (XO) in the midbrain. The result was then expressed as μmol/mg protein and μmol/h/mg protein, respectively (Montgomery 1961). The reduction of DTNB by GSH was used for the estimation of glutathione reductase activity. The GPx activity was determined as μmol of GSH oxidized/min/mg protein (Paglia and Valentine 1967; Goldberg and Spooner 1983). The activity of SOD in the samples was measured and expressed in U/mg protein (Nishikimi et al. 1972). Finally, the activity of GST was quantified by measuring the absorbance of the sample at a wavelength of 310 nm and expressed as mmol/min/mg (Habig et al. 1974).

### Histopathological examination

Midbrain tissues were kept in 10% formalin buffered solution (pH 7.4), rinsed with saline, and fixed at 56 ℃ for 24 h in a hot air oven before being paraffinized. 4.5 μm sections were cut from paraffin blocks, placed on glass slides, deparaffinized, and stained with Mayer's hematoxylin and eosin (H&E) stain (Levison et al. 1996). The stained sections were examined with light microscopes and images were photographed with a digital camera (Nikon Corporation Co., Ltd., Japan).

### Molecular docking simulation against PP2A, α-syn, and TH

The binding sites of PP2A, α-syn, and TH were obtained from the protein data bank (PDB codes: α-syn(1XQ8), PP2A (2IAE), and TH (AF-Q15587-F1)) available on http://www.rcsb.org/pdb or Uniprot. First, water molecules were removed from the complex, and then protein preparation options were used to correct crystallographic disorders and unfilled valence atoms. The protein energy was minimized using CHARMM force fields. Chem-Bio Draw Ultra17.0 was utilized to create 2D structures of MTF, BBR, and CAF compounds, which were saved in MDL-SD file format. The MDL-SD files were then opened with MOE 2016 software, protonated to generate 3D structures, and energy was minimized using the MMFF94 force field and 0.1 RMSD kcal/mol. The docking scores of the best-fitted poses with the active site were recorded and a 3D view was visualized by Discovery Studio 2019 Client software.

### Docking against α-syn Ser129, PLK, CK2, and GRK

The 3D structures of CAF, BBR, and MTF were retrieved from PubChem with their respective CIDs: 2519, 2353, and 4091. The crystal structures of the target proteins, α-syn, polo-like kinase (PLK), casein kinase 2 (CK2), and G protein-coupled receptor kinase (GRK) were obtained from the RCSB Protein Data Bank using the PDB IDs 1XQ8, 4I5P, 8Q9S, and 6PJX, respectively. Protein structures were prepared using AutoDock Tools, involving the removal of water molecules, the addition of hydrogen atoms, and the assignment of Kollman charges. Binding sites for PLK, CK2, and GRK were defined based on the coordinates of their respective co-crystal ligands. For α-syn, the docking grid box was centered on the Ser129 residue, and additional blind docking simulations were performed to explore the broader binding potential. Redocking of the co-crystal ligands was conducted to validate the docking protocol, with root-mean-square deviation (RMSD) values calculated to assess accuracy. Molecular docking simulations were performed using GNINA 1.0.1 (McNutt et al. 2021). The molecular docking protocol modeled flexible ligands interacting with rigid protein targets. Molecular visualizations of the docking poses were generated using Discovery Studio Visualizer 2021.

### Statistical analysis

Results from each group are presented as a mean ± standard error of the mean (SEM). One-way ANOVA and Tukey's multiple comparison post hoc test were utilized to assess the differences between various groups. Statistical analyses were conducted utilizing GraphPad Prism (version 8.0.1) (San Diego, CA, USA) and IBM SPSS Statistics 25 software (IBM, USA). The statistical threshold for "significant" was set at *p* < 0.05.

## Results

### Berberine and caffeine combined therapy inhibited weight loss in rats induced by ROT

**Fig. 2:**
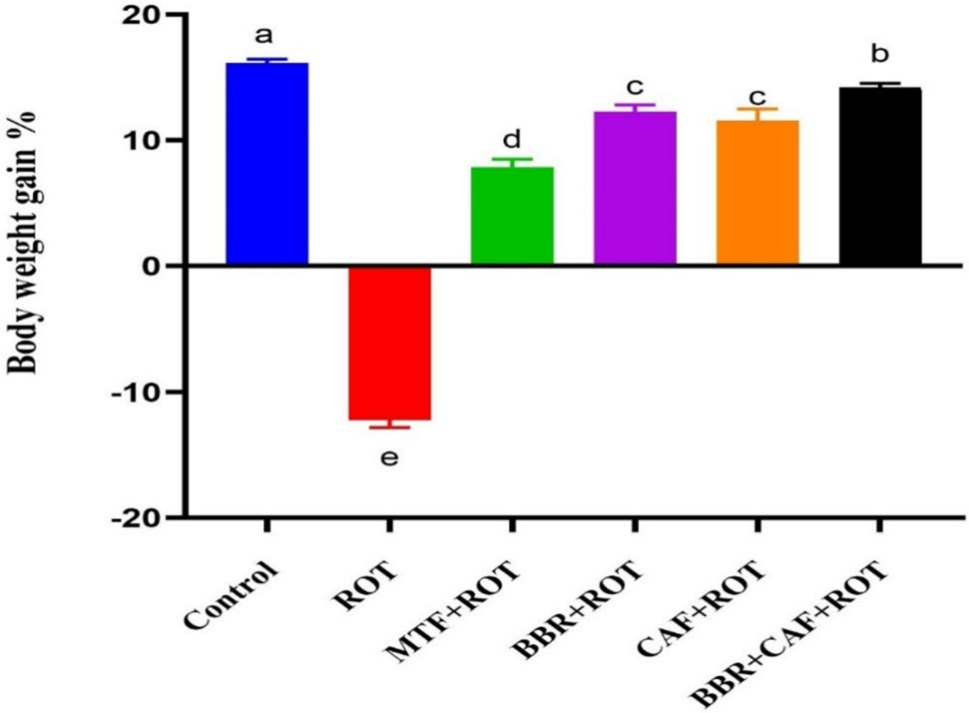
Effects of different treatments on the percentage of body gain in the different experimental rat groups. Data are expressed as mean ± SEM (*n* = 6) and evaluated using one-way ANOVA with Tukey’s post hoc test; means indicated by different letters are significantly different (*p* < 0.05), with the highest value designated as (a) and the lowest value as (d). *ROT* rotenone, *MTF* metformin, *BBR* berberine, *CAF* caffeine

The percentage of body weight gain between days 0 and 28 of the study was measured (Fig. 2). The ROT-induced animals had considerable body weight loss compared to control rats (*p* < 0.05). Monotherapy with BBR, CAF, and MTF resulted in increased body weight gain compared to ROT-induced rats (*p* < 0.05). The combination of BBR and CAF demonstrated the most significant enhancement in body weight increase compared to monotherapy, with values approaching the control level.

### Berberine and caffeine combined treatment enhanced motor functions in pole descending and holding bar test

**Fig. 3:**
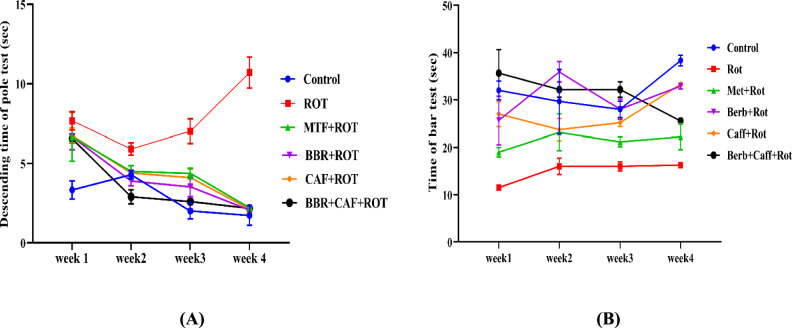
Effect of different treatments of ROT-treated rats for four weeks on motor function. **A** Pole descending test and **B** bar holding test. The data were expressed as mean ± SEM. *ROT* rotenone, *MTF* metformin, *BBR* berberine, *CAF* caffeine

A 4-week pole descending and bar holding tests were carried out to examine the effects of different treatments of ROT-induced rats on motor function. Compared to the control rats, the ROT-induced rats had a significant increase in the time taken by rats to reach the base of the pole in week four versus week one indicating typical locomotor symptoms of (PD). Treatment with BBR and/or CAF as well as MTF decreased pole descending time significantly as compared to ROT-induced rats (Fig. 3A). In the bar holding assay, the ROT-induced group showed a significant decrease in time in week 4 of the study versus week 1 compared to the control group. MTF administration had no significant effect when compared to ROT-induced rats. However, BBR and/or CAF treatments increased the time of a holding bar compared to the induced group (*p* < 0.05) (Fig. 3B).

### Combined berberine and caffeine improved dopaminergic-related markers in the experimental rats

**Fig. 4:**
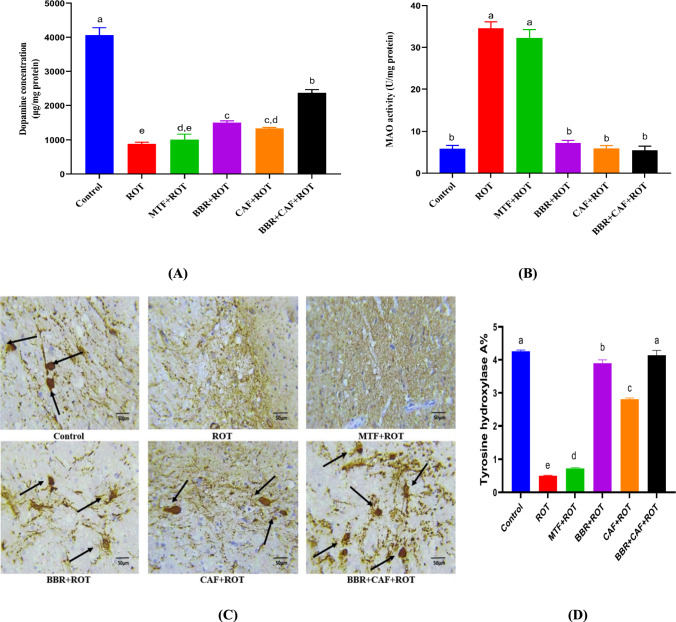
Effects of different treatments on dopaminergic-related biomarkers. **A** Dopamine levels, **B** MAO activities, **C** immunohistochemistry of TH, and **D** quantitative analysis of TH of degenerated neurons in the midbrain. Data are expressed as mean ± SEM (*n* = 6) and evaluated using one-way ANOVA with Tukey’s post hoc test; means indicated by different letters are significantly different (*p* < 0.05), with the highest value designated as (a) and the lowest value as (e). *ROT* rotenone, *MTF* metformin, *BBR* berberine, *CAF* caffeine, *TH* tyrosine hydroxylase, *MAO* monoamine oxidase

The DA level was considerably reduced in the ROT-induced group relative to the control group (*p* < 0.05). Treatment with BBR and/or CAF resulted in a significant boost in DA levels in comparison to ROT-induced animals (*p* < 0.05), while the MTF exhibited a non-significant elevation in DA levels relative to ROT-induced animals (*p* < 0.05). The combination administration of BBR and CAF demonstrated the highest DA levels, approaching the control value (*p* < 0.05), in contrast to monotherapy (Fig. 4A). Conversely, MAO activity was markedly elevated in the ROT-induced rats above that of the control group (*p* < 0.05). MAO activity was normalized after treatment with BBR and/or CAF in comparison to ROT-induced rats (*p* < 0.05). However, the standard drug MTF exhibited no notable effect on MAO activity in comparison to ROT-induced rats (Fig. 4B). At the histopathological assessment level, the ROT-induced rats exhibited lower levels of TH than the control group (*p* < 0.05). The BBR and/or CAF treatments restored normal levels of TH relative to the control rats (*p* < 0.05). The MTF exhibited a non-significant alteration in TH levels relative to ROT-induced animals (Fig. 4C, D).

### Berberine and caffeine combination therapy prevented the increase in total and α-Syn p^ser129^ in the midbrain of experimental rats

**Fig. 5:**
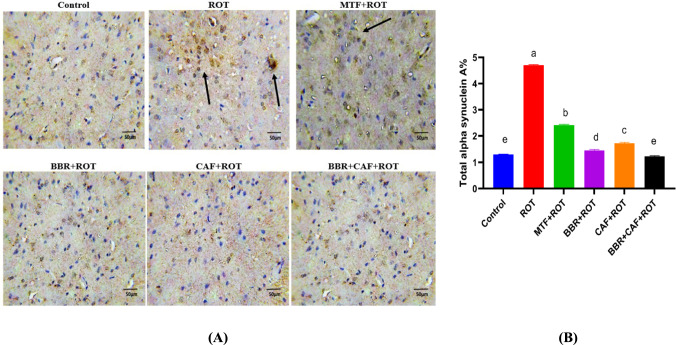

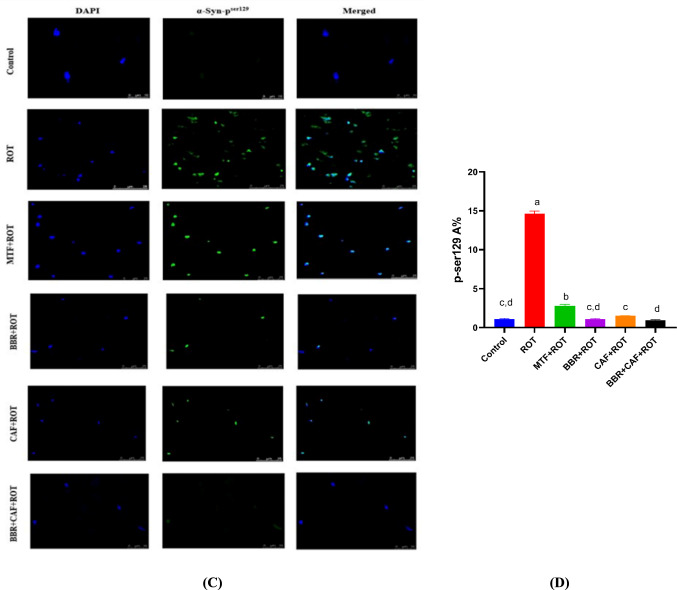
Effects of different treatments on total α-Syn and α-Syn-p^ser129^ levels in midbrain of experimental rats. **A** Immunohistochemistry of total α-syn, **B** quantitative analysis of total α-syn in the midbrain, **C** immunofluorescence of α-syn-p^ser129^, and **D** quantitative analysis of α-syn-p^ser129^ in the midbrain. Data are expressed as mean ± SEM (*n* = 6) and evaluated using one-way ANOVA with Tukey’s post hoc test; means indicated by different letters are significantly different (*p* < 0.05), with the highest value designated as (a) and the lowest value as (e). *ROT* rotenone, *MTF* metformin, *BBR* berberine, *CAF* caffeine, *α-syn* alpha-synuclein

The total α-Syn and α-Syn-p^ser129^ proteins in the midbrain were determined by immunohistochemistry and immunofluorescence. At immunohistopathological levels, the total α-Syn levels in the midbrain of ROT-induced rats were significantly elevated relative to control rats (*p* < 0.05). Administration of MTF as well as BBR and/or CAF declined the α-Syn aggregation compared to ROT-induced rats (*p* < 0.05; Fig. 4A, B). Moreover, the α-Syn-p^ser129^ levels were highly elevated in the ROT-induced rats than in the control group (*p* < 0.05). Conversely, the administration of MTF as well as BBR and/or CAF displayed a marked decline in α-Syn-p^ser129^ protein levels compared to ROT-induced rats (*p* < 0.05; Fig. 5C, D).

### Berberine and caffeine combination significantly restored PP2A and AMPK normal levels in the midbrain of experimental rats

**Fig. 6:**
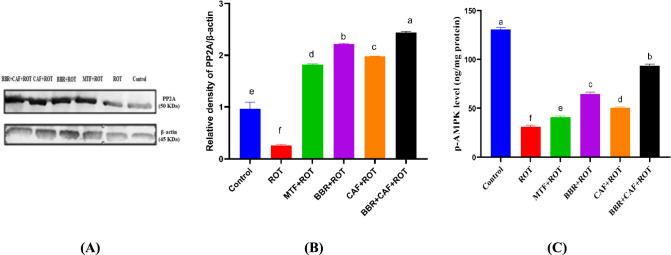
Analysis of protein phosphatase 2A and AMPK levels in midbrain of experimental rats. **A** Western blot of PP2A and β-actin proteins, **B** quantification of the relative density of PP2A/β-actin, and **C** ELISA levels of p-AMPK. Data are expressed as mean ± SEM (*n* = 3) and evaluated using one-way ANOVA with Tukey’s post hoc test; means indicated by different letters are significantly different (*p* < 0.05), with the highest value designated as (a) and the lowest value as (f). *ROT* rotenone, *MTF* metformin, *BBR* berberine, *CAF* caffeine, *PP2A* protein phosphatase 2A, *p-AMPK* adenosine monophosphate-activated protein kinase

As shown in Fig. 6A, B, in comparison to the control group, the ROT-induced group exhibited reduced levels of PP2A protein expression (*p* < 0.05). Treatment with MTF as well as BBR and/or CAF exhibited a notable restoration of PP2A protein expression levels compared to the ROT-induced animals. Further, the activated p-AMPK level showed a significant decrease in the ROT-induced group versus the control (*p* < 0.05). Administration of MTF, as well as BBR and/or CAF, restored p-AMPK level to normal compared to ROT-induced groups (*p* < 0.05; Fig. 6C).

### Berberine and caffeine combination therapy reduced neuroinflammatory cytokines levels in the midbrain of experimental rats

**Fig. 7:**
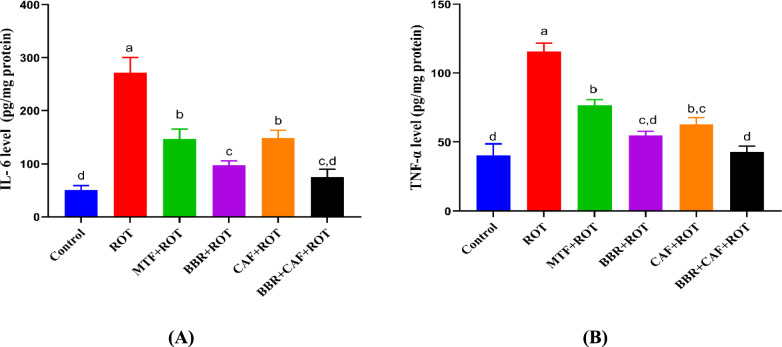
Levels of neuroinflammatory cytokines in the different experimental rats’ groups. **A** IL-6 and **B** TNF-α. Data are expressed as mean ± SEM (*n* = 6) and evaluated using one-way ANOVA with Tukey’s post hoc test; means indicated by different letters are significantly different (*p* < 0.05), with the highest value designated as (a) and the lowest value as (d). *ROT* rotenone, *MTF* metformin, *BBR* berberine, *CAF* caffeine

Dysregulations in brain tissue and peripheral organs activate pro-inflammatory signaling pathways that contribute to the neurotoxicity linked to PD, correlating with disease severity and disability. Neuroinflammation-associated cytokines, IL-6 and TNF-α, were elevated significantly in the ROT-induced group versus the control group (*p* < 0.05). The pre-treatment with MTF as well as BBR and/or CAF showed a significant diminish in the levels of IL-6 and TNF-α compared to the ROT-induced group (*p* < 0.05). The combination of BBR and CAF showed a potential advantage in mitigating neuroinflammation by decreasing the levels of IL-6 and TNF-α to approximately control values (Fig. 7A, B).

### Berberine and caffeine combination therapy decreased oxidative markers and enhanced antioxidant parameters in the midbrain of experimental rats

Dopaminergic neurons undergo degeneration due to disruptions in the physiological maintenance of the redox balance in the neurons. In the ROT-induced group, MDA and NO levels, together with XO activity, were considerably elevated, whereas GSH, GPX, GST, and SOD levels were diminished in comparison to the control group (*p* < 0.05). BBR and/or CAF treatments significantly reduced MDA and NO levels as well as XO activity (*p* < 0.05) and considerably augmented the activities of antioxidant enzymes relative to the ROT-induced group (*p* < 0.05). The combined therapy with BBR and CAF demonstrated a potential improvement in oxidant and antioxidant levels relative to other therapies, with values comparable to control levels (Table 1).

**Table 1:** Levels of oxidative markers in the different experimental rats’ groups

Groups	MDA (nmol/mg protein)	NO (μmol/mg protein)	XO (μmol/h/mg protein)	GSH (mg/mg protein)	GPX (U/mg protein)	GST (mmol/min/mg protein)	SOD (U/mg protein)
Control	23.8 ± 1.8^e^	1.1 ± 0.1^c^	1.5 ± 0.2^e^	89.4 ± 5.2^a^	132.4 ± 5.9^a^	6.3 ± 0.3^a^	749.9 ± 9.4^a^
ROT	112.5 ± 0.8^a^	4.3 ± 0.2^a^	12.3 ± 0.1^a^	21.3 ± 3.0^d^	19.4 ± 1.7^e^	1.4 ± 0.2^d^	196.1 ± 1.2^e^
MTF + ROT	75.2 ± 2.8^b^	2.5 ± 0.2^b^	10.2 ± 0.5^b^	32.7 ± 4.5^c, d^	27.9 ± 3.3^d,e^	2.6 ± 0.3^c,d^	270.1 ± 1.4^d^
BBR + ROT	45.1 ± 1.5^d^	1.8 ± 0.3^b, c^	4.0 ± 0.1^d^	67.5 ± 2.4^b^	56.9 ± 2.0^c^	3.7 ± 0.3^b,c^	483.3 ± 5.8^c^
CAF + ROT	58.4 ± 0.5^c^	2.3 ± 0.5^b, c^	7.5 ± 0.5^c^	41.8 ± 2.2^c^	50.2 ± 5.1^c,d^	2.8 ± 0.2^c,d^	311.2 ± 2.4^d^
BBR + CAF + ROT	22.2 ± 1.7^e^	1.5 ± 0.2^b, c^	3.8 ± 0.2^d^	72.2 ± 1.1^b^	93.1 ± 9.5^b^	4.7 ± 0.5^b^	548.5 ± 3.7^b^

Data are expressed as mean ± SEM (*n* = 6) and evaluated using one-way ANOVA with Tukey’s post hoc test; means indicated by different letters are significantly different (*p* < 0.05), with the highest value designated as (a) and the lowest value as (e)

*ROT* rotenone, *MTF* metformin, *BBR* berberine, *CAF* caffeine

### Berberine and caffeine combination therapy preserved the normal histoarchitecture in the midbrain of experimental rats

**Fig. 8:**
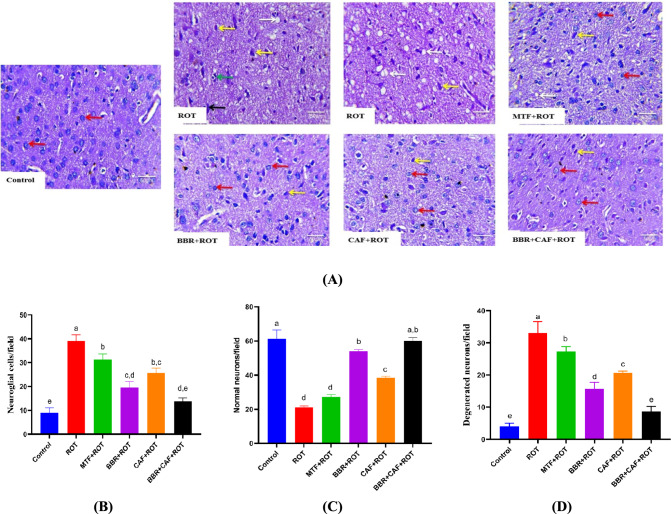
Illustrative photomicrographs of rat midbrain sections stained with H&E.** A** Hematoxylin and eosin photomicrographs, red arrows indicate normal neuron histoarchitecture. Yellow arrows indicate dark-stained, shrinking neurons. Satellitosis-related necrotic neurons are shown by the black arrow. The green arrow shows neurophagia. White arrows indicate neuropil vacuolation and enlarged pericellular vacuolation (×400), **B** quantitative analysis of neuroglial cells per field in the midbrain, **C** quantitative analysis of normal cells per field in the midbrain and, **D** quantitative analysis of degenerated neurons per field in the midbrain. Data are expressed as mean ± SEM (*n* = 6) and evaluated using one-way ANOVA with Tukey’s post hoc test; means indicated by different letters are significantly different (*p* < 0.05), with the highest value designated as (a) and the lowest value as (e). *ROT* rotenone, *MTF* metformin, *BBR* berberine, *CAF* caffeine, PP2A

Midbrain sections were stained with hematoxylin and eosin in the different experimental rats’ groups (Fig. 8). Histological examination of the midbrain sections from the control rats showed typical tissue architecture. ROT-induced rats exhibited deteriorated, shrunken, darkly stained neurons that were necrotic and linked with satellitosis and neuronophagia, as well as few degenerated neurons and enlarged pericellular spaces and neutrophil vacuolation (Fig. 8B–D). In addition, MTF-treated rats displayed a small improvement in the midbrain with deteriorated, shrunken, darkly stained neurons that were necrotic and linked with satellitosis and neuronophagia. The administration of BBR and/or CAF showed marked histoarchitecture enhancement with normal neurons and few degenerated neurons.

### Molecular docking analysis against α-syn, PP2A, and TH

**Fig. 9:**
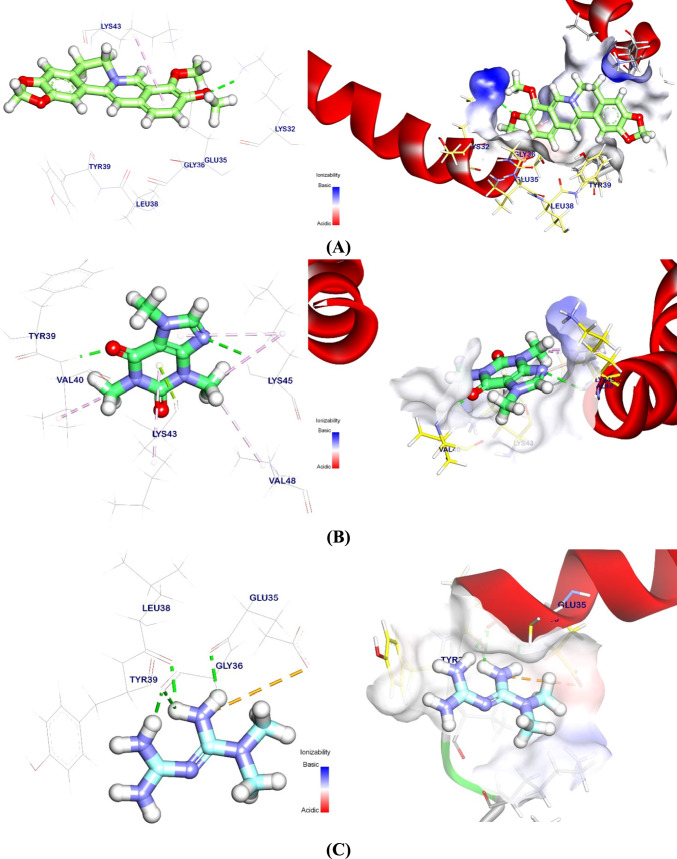

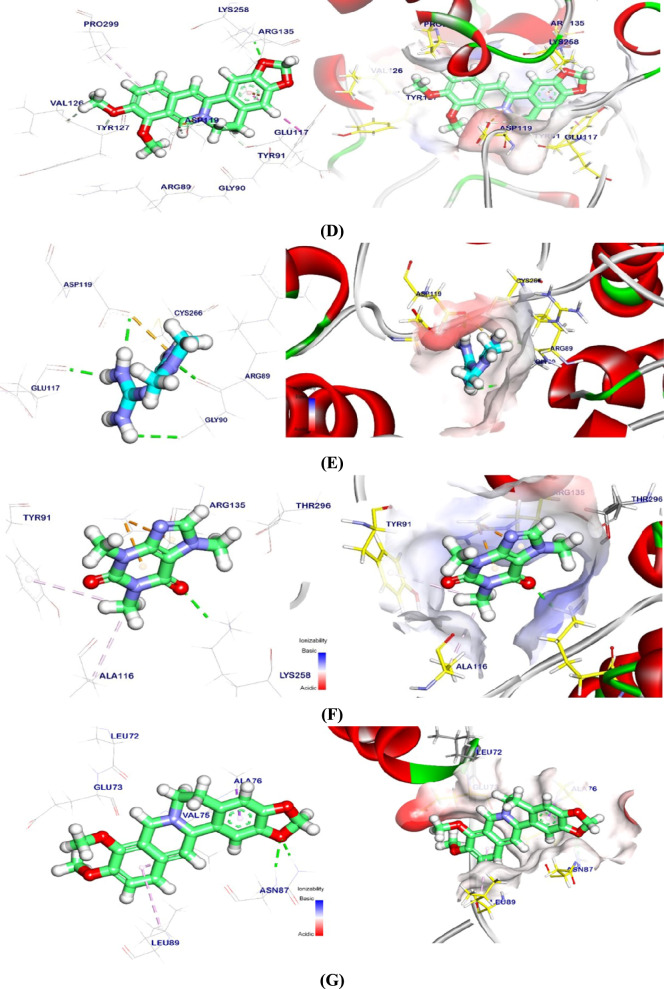

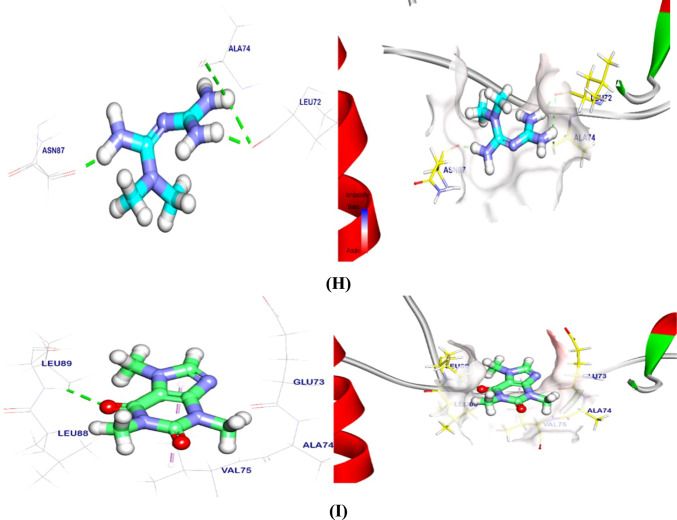
3D and surface mapping of MTF, CAF, and BBR against target sites. **A** BBR against α-syn, **B** MTF against α-syn, **C** CAF against α-syn, **D** BBR against PP2A, **E** MTF against PP2A, **F** CAF against PP2A, **G** BBR against TH, **H** MTF against TH, and **I** CAF against TH. *ROT* rotenone, *MTF* metformin, *BBR* berberine, *CAF* caffeine, *α-syn* alpha-synuclein, *PP2A* protein phosphatase 2A, *TH* tyrosine hydroxylase

The binding energy of the BBR binding mode against the α-syn target site was − 3.21 kcal/mol. With Lys43, one hydrophobic π-interaction was noted. It also formed a hydrogen bond with Lys32 that was 2.55 Å bond length (Fig. 9A). The binding mode of CAF at the α-syn target site exhibited a binding energy of − 5.05 kcal/mol. Five Pi-Alkyl and Pi-lone pair interactions were established between CAF and Val48, Lys45, Val40, and Met43. Furthermore, two hydrogen bonds with lengths of 2.84 and 2.61 Å were established between CAF and Lys45 and Val40 (Fig. 9B). Docking of MTF exhibit a binding energy of − 4.04 with α-syn. It interacted with Gly36, Leu38, and Glu35 by four hydrogen bonds with lengths of 2.08, 2.65, 2.26, and 2.22. However, Glu35 formed an attractive interaction with cationic amine in MTF (Fig. 9C). Also, regarding the binding with PP2A, BBR interacted with Arg135, Tyr91, and Pro299 at 2.18 Å (Fig. 9D). CAF generated four Pi-Alkyl/Pi-cation interactions with Arg135, Tyr91, Ala116, and a 2.39-Å hydrogen bond with Lys258 (Fig. 9E). MTF interacts with phosphatase 2A, creating three hydrogen bonds and one ionic contact with Arg89, Gly90, Glu117, and Asp119 at 2.44, 3.00, 2.15, and 2.11 Å (Fig. 9F). For TH binding, BBR binding affinity showed − 5.41 kcal/mol. BBR interacted with Ala76, Leu89, Val75 Pi-Alkyl, and Pi-sigma. Asn87 had a 3.38-Å hydrogen bond (Fig. 9G). CAF had one Pi-Alkyl interaction with Val75 and one hydrogen bond with Leu89 at 2.61 Å (Fig. 9H). Finally, MTF hydrogen bonded with Asn87, Leu72, and Ala74 at 1.97, 1.99, 2.47, and 3.00 Å for TH (Fig. 9I). In addition, DG, RMSD, and interactions of tested ligands against target sites are shown in Table 2.

**Table 2:** Displays (DG, RMSD, interactions) kcal/mol of evaluated ligands versus target locations

Targets	Tested compounds	RMSD value (Å)	Docking (affinity) score (kcal/mol)	Interactions	
				H.B	Pi-interaction
α-syn	BBR	1.79	− 3.21	–	–
	CAF	1.57	− 5.05	2	4
	MTF	1.39	− 4.04	4	–
PP2A	BBR	1.34	− 6.54	1	4
	CAF	1.49	− 4.88	1	4
	MTF	0.65	− 4.11	4	–
TH	BBR	0.95	− 5.41	1	3
	CAF	1.70	− 4.10	1	1
	MTF	1.17	− 4.05	4	–

### Molecular docking analysis against α-syn Ser129, PLK, CK2, and GRK

The molecular docking results against α-syn reveal that BBR exhibits the highest binding affinity (− 5.11 kcal/mol) for the protein pocket surrounding the Ser129 residue of α-syn, followed by CAF (− 3.26 kcal/mol) and MTF (− 3.23 kcal/mol). Berberine's superior binding interaction is further corroborated by its higher CNN affinity score (4.31) compared to CAF (3.07) and MTF (2.69), indicating a more favorable binding pos. Notably, among the tested compounds, CAF was the only one to form a direct interaction with the Ser129 residue, albeit a weak van der Waals interaction. Neither BBR nor MTF showed direct engagement with Ser129. Additionally, blind docking of CAF with α-syn did not identify strong binding affinities for other pockets of the protein, suggesting limited interaction potential beyond the defined region. On the other hand, blind docking of BBR with the protein generated nine poses (with affinities ranging from − 4.86 to − 5.14 kcal/mol) where none of them bound to Ser129 residue (Supplementary Fig. S1A–C).

Additionally, polo-like kinase (PLK) is a key enzyme implicated in phosphorylating α-syn at the Ser129 residue, a modification linked to pathological aggregation in neurodegenerative diseases. Among the tested compounds, BBR exhibited the strongest binding affinity toward the active site of PLK (− 8.49 kcal/mol), surpassing CAF (− 5.90 kcal/mol) and MTF (− 3.87 kcal/mol). Berberine’s high CNN affinity (6.34) further underscores its potential as a potent inhibitor of PLK. In terms of interactions, BBR formed several critical interactions like hydrogen bond and hydrophobic interactions with residues in the active site, stabilizing its binding pose. Notably, π–π stacking interactions were observed between BBR’s aromatic rings and key active site residues, further enhancing its binding stability. In comparison, CAF primarily engaged in hydrophobic interactions and hydrogen bond, while MTF exhibited minimal interactions, mostly through a hydrogen bond and π–sigma interaction (Supplementary Fig. S2A–C). The co-crystal ligand validated the docking protocol with the highest binding affinity of − 8.58 kcal/mol, a CNN pose score of 0.97, and an RMSD value of 0.64 nm, confirming the reliability of the docking approach (Supplementary Fig. S3).

Moreover, casein kinase 2 (CK2) is another critical enzyme involved in phosphorylating α-syn at Ser129, contributing to its pathological aggregation in neurodegenerative diseases. Berberine demonstrated an exceptional binding affinity toward the CK2 active site (− 9.09 kcal/mol), outperforming CAF (− 5.78 kcal/mol) and MTF (− 4.29 kcal/mol). Berberine's high CNN affinity score (6.84) and moderate CNN pose score (0.77) suggest strong and stable interactions with the enzyme. Analysis of interaction types revealed that BBR forms a network of stabilizing interactions with CK2, including multiple π–sigma interactions with key residues in the active site and hydrophobic contacts that enhance binding stability. These interactions collectively contribute to its robust binding profile. In comparison, CAF primarily engages in hydrophobic, π–sulfur interactions and a few hydrogen bonds, while MTF shows limited interaction potential, relying mainly on hydrogen bonding (Supplementary Fig. S4A–C). The co-crystal ligand validated the docking methodology with the highest binding affinity (− 10.19 kcal/mol), an RMSD of 0.30 nm, a CNN affinity of 8.10, and an excellent CNN pose score of 0.99 (Supplementary Fig. S5).

 Finally, BBR demonstrated the highest binding affinity (-11.06 kcal/mol) for GRK, an enzyme that is also associated with α-syn phosphorylation. This was significantly higher than that of CAF (-6.44 kcal/mol) and MTF (-5.02 kcal/mol). Berberine's high CNN affinity (6.91) and moderate pose score (0.70) suggest robust and specific interactions with the active site of the enzyme (Supplementary Fig. S6A–C). Interestingly, the co-crystal ligand showed a weaker binding affinity (− 8.85 kcal/mol) than BBR (Supplementary Fig. S7A–C). Overall, the molecular docking results are shown in Supplementary Table S1.

## Discussion

Parkinson's disease is defined as progressive motor and non-motor impairments that exhibit several neuropathological mechanisms such as dopaminergic neuron loss, mitochondrial failure, oxidative stress, neuroinflammation, and aggregation of α-syn (Poewe et al. 2017). Recently, some researchers have focused on rotenone administration to develop PD model in rodent since it resembles PD's chronic development offering insights into the specific susceptibility of nigrostriatal degeneration. This model facilitates the study of several molecular and metabolic mechanisms that explain PD pathogenesis and its clinical symptoms (Lama et al. 2021). Rotenone is a lipophilic inhibitor of mitochondrial complex I that penetrates the blood–brain barrier and cellular membranes. Administration of rotenone into animal models induces selective acute toxicity in dopaminergic cells through abnormal α-syn aggregation, mitochondrial oxidative stress, inflammation, and cell death resulting in development of PD in rats and primates (Van der Brug et al. 2015).

The conventional treatments for PD that are currently available are costly and result in adverse effects. Therefore, it is imperative to investigate innovative therapeutic strategies that target the biochemical pathways that result in neuronal loss and dysfunction. Repurposing multifunctional pharmaceuticals such as MTF may safeguard PD neuronal cells. However, MTF may worsen PD neuropathology by causing hyperhomocysteinemia that induces the onset of oxidative stress, mitochondrial failure, apoptosis, and endothelial dysfunction as well as folate and B12 deficiencies (Shurrab and Arafa 2020). Natural resources have long been valued as potential treatment of PD due to their anti-inflammatory and anti-oxidative effects, roles in mitochondrial homeostasis, and other neuroprotective mechanisms. Although natural alkaloids and MTF possess distinct structures, they exhibit numerous similarities in their mechanisms of action, suggesting both could serve as potential protection and treatments for neurodegenerative disorders. Previous studies have demonstrated that each BBR or CAF monotherapy had neuroprotective effects in PD in vivo experimental models (Adeyeye et al. 2023; Tseng et al. 2024).

Multi-target drugs are replacing chemical monotherapy due to the ineffectiveness, resistance problems, and adverse effects of single synthetic medications in progressive neurodegenerative disorders such as PD. Synergistic effects might arise when more than one chemical compound from plant extracts interacts with distinct targets resulting in increased pharmacological actions (David et al. 2015). Nonetheless, no prior studies have utilized the combination of caffeine and berberine in experimental models of neurodegenerative diseases such as PD. Therefore, the present study examined the neuroprotective effects of BBR and CAF pre and co-treatment, both alone and in combination, as a novel strategy relative to MTF administration in a ROT-induced rat model of PD.

Fatigue and weight loss are likely common in PD patients due to diminished energy intake resulting from anorexia, dysphagia, and gastrointestinal dysfunction. In addition, rigidity and tremors may increase energy expenditure resulting in losing weight (Okuma 2012). In the current research, we assessed the body weight gain/loss percentage as well as the modified motor parameters by utilizing two distinct tests in rats from various experimental groups for a period of four weeks. The current study found that ROT administration significantly decreased the body weight of rats. Furthermore, we noted that the duration for rats to descend the pole test considerably increased and the time spent grasping the bar diminished following ROT injection over the four weeks. These results align with previous research that has demonstrated that ROT-induced deficits influence motor functions (Ibarra-Gutiérrez et al. 2023). The current study demonstrated that the administration of BBR and CAF prevented weight loss and motor function deficits caused by ROT treatment compared to MTF treatment, supporting previous research findings (Adeyeye et al. 2023; Tseng et al*.* 2024), indicating enhanced welfare of PD model.

Dopamine is a key neuromodulator that controls voluntary movements and cognition, and its deficiency has been linked to PD pathogenesis (Klein et al. 2019). MAO enzymes are expressed in the striatum and they are involved in the oxidative deamination of DA neurotransmitters. MAO-B enzyme isoform degrades DA; therefore, it worsens PD's symptoms and prolongs its effect (Tan et al. 2022). Preceding studies have established that BBR elevates the levels of brain neurotransmitters, including DA by decreasing the activities of MAOs (Och et al. 2022). Furthermore, it has been shown that the inhibition of MAO-B by CAF may promote an elevation in DA levels, hence mitigating motor issues (Zhou et al. 2019). Our results showed that the pre-treatment with BBR and CAF combination, as opposed to each one alone, showed a considerable rise toward restoring normal DA levels with a notable decrease in MAO activity. TH serves as the rate-limiting factor in catecholamine production and is essential in DA biosynthesis. PD may be seen as a condition characterized by a deficiency of striatal TH (Nagatsu et al. 2019). The results of the current study indicate that the immunostaining pars compacta cells exhibited a significant reduction in brownish immunoreactive staining following the administration of ROT. Co-administration of BBR and CAF markedly restored the TH level (Karuppagounder et al. 2021; Sunhe et al. 2024).

Several lines of evidence revealed that α-syn is a critical factor in the pathophysiology of PD. In Lewy bodies, 90% of α-syn is phosphorylated at Serine 129. However, the typical brain phosphorylates 4% or fewer of the total α-syn at this location (Anderson et al. 2006; Kawahata et al. 2022). It has been proposed that samples of PD patients exhibit elevated total and pS129 α-syn levels, as well as pS129/total α-syn ratios in the cerebrospinal fluid (Constantinides et al. 2021), plasma (Lin et al. 2019), and erythrocyte (Tian et al. 2019). Increased α-synuclein levels have been demonstrated to inhibit the TH enzyme, which is essential for DA synthesis. Moreover, preliminary data suggest that the phosphatase enzyme may become less active due to conformational changes in the aggregated α-syn, potentially affecting its ability to bind the PP2A catalytic subunit (Wu et al. 2012). Earlier studies have focused on utilizing combined phytochemicals to target aggregated α-syn in PD models (Javed et al. 2019). Consistent with our findings, the combined therapy of BBR and CAF exhibited a significant modulatory effect on the inhibition of α-syn aggregation and the enhancement of PP2A levels in PD model.

There is a correlation between PD, low-grade chronic inflammation, and redox imbalance (Pajares et al. 2020). Microglial activation may be induced by central nervous system (CNS) insults, including environmental or genetic influences that potentially aggravate pathogenesis, resulting in increased levels of pro-inflammatory cytokines including TNF-α and IL-6, as well as reactive oxygen species and gradual neuronal death. The release of inflammatory mediators is triggered by excessive oxidative stress, which simultaneously activates an inflammatory response. This inflammatory cascade is thought to be a major mechanism behind the loss of neurons and the advancement of PD (Dadgostar et al. 2022). It has been previously established that inflammation facilitates α-syn aggregation. Microglia release exosomes and pro-inflammatory cytokines that spread insoluble α-syn to normal cells, leading to rapid aggregation of pathogenic α-syn (Xia et al. 2019). Furthermore, recent studies have shown that inflammation can diminish the synthesis, packing, and discharge of DA, so impairing or undermining the effectiveness of conventional antidepressant therapy (Gopinath et al. 2023; Zhang et al. 2024). Inflammation-induced DA depletion may result in symptoms of depression and psychomotor slowness (Felger 2017). Our research revealed increased neuroinflammatory cytokines levels in the midbrain of ROT-induced rats, which is in line with other findings and suggests that neuroinflammatory processes play a role in ROT-induced neurotoxicity and motor dysfunction (Tseng et al. 2024).

Current PD therapies target is to restore the normal DA levels by administration of DA precursors and agonists or preventing endogenous breakdown and in addition to treatment of some PD symptoms. On the other side, herbal medicine studies may assist as a neuroprotective approach since inflammation is a crucial path mechanism in PD. Several lines of evidence indicated that CAF may exert an anti-neuroinflammatory impact (Ren and Chen 2020). In adult mouse brains, CAF daily administration prevents lipopolysaccharide (LPS)-induced neuroinflammation and synaptic dysfunction (Badshah et al. 2019). In adult mouse brains, daily intraperitoneal injection of CAF may prevent LPS-induced oxidative stress and neuroinflammation by controlling Nrf2/TLR4 and dose dependently decreases the microglia activation in three hippocampal regions (Brothers et al. 2010). It may regulate microglia-mediated neuroinflammatory response linked to PD as a crucial neuroprotective element (Madeira et al. 2017). In the MPTP animal model, daily intraperitoneal CAF administration diminishes microglial activation and prevents disruption of the blood–brain barrier, which results in less loss of dopaminergic neurons (Chen et al. 2013).

Recent studies showed that the diverse pharmacological capabilities of BBR, including its anti-inflammatory, antibacterial, antiviral, anti-diabetic, anti-diarrheal, and anti-dyslipidemic activities (Gasmi et al. 2024). Through an analysis of midbrain inflammatory cytokines, we identified the anti-inflammatory properties of BBR, CAF, and MTF. Our current therapies resulted in a substantial decrease in TNF-α and IL-6 levels, with the highest inhibition observed in the groups treated with combined BBR and CAF, suggesting their potential neuroprotective properties in the midbrain to mitigate behavioral deficits induced in the PD model.

Oxidative stress is considered as a hallmark of the neurodegenerative pathogenesis of brain aging-related dysfunction and is a crucial factor in the etiology of Parkinson's disease. ROS and RNS may contribute to dopaminergic neuronal damage and death since evidence suggests the presence of oxidative stress in early-stage PD patients (Ferrer et al*.* 2011). Dopaminergic neurons are especially vulnerable to oxidative stress in PD because of the presence of ROS-producing enzymes such as MAOs (Reiter 1995). Previous research has shown a robust link between ROT-induced motor dysfunction and changes in striatal nitrosative and oxidative conditions (Mansour et al. 2018). Our outcomes in parallel revealed noticeably increased striatal MDA and NO levels besides XO activity and decreased antioxidant balance in ROT-induced rats. In the current work, we evaluated the promising antioxidant effect of BBR and/or CAF compared to MTF. BBR is a strong antioxidant that may be utilized as an antioxidant treatment for neurological illnesses because it can prevent the production of ROS and preserve the integrity and function of the mitochondrial membrane (Tseng et al. 2024). CAF has been shown to inhibit lipid peroxidation by lowering the generation of ROS. It has been demonstrated that CAF protects dopaminergic neurons by triggering pathways that prevent oxidative damage, including nuclear factor erythroid 2-related factor 2-Keap1 and peroxisome proliferator-activated receptor gamma coactivator 1-α (Zhou et al. 2019). CAF can also act as an antioxidant by increasing GST activity (Schepici et al. 2020). Interestingly, we found that the combined treatment of BBR and CAF considerably raised the antioxidant enzyme levels in rats when compared to the other groups of rats treated with BBR, CAF, or MTF, indicating the synergistic protective actions.

In research trials and clinical investigations, dosage conversions and the establishment of a safe dose for humans are critical elements. Numerous methodologies exist for establishing the first-in-human dosage, which serves as a valuable reference for researchers involved in preclinical and early clinical drug development to estimate the human equivalent dose from animal models. The animal doses of CAF and BBR in the current study can be translated to human equivalent doses with the method provided by Reagan-Shaw et al. (2008): the human equivalent dose (mg/kg) is calculated by multiplying the rat dose (mg/kg) by the ratio of the rat Km factor to the human Km factor, with the Km factor being 6 for rats and 37 for humans. Consequently, 5 mg/kg × 6/37 × 70 = 57 mg daily represents the dosage of CAF for an average individual weighing 70 kg, corresponding to the amounts employed in our study. An individual weighing 70 kg would need 567.6 mg of BBR daily, calculated as 50 mg/kg × 6/37 × 70, according to the levels employed in our investigation. The combined therapies' doses were calculated to be 28.4 mg for CAF and 284 mg for BBR.

Molecular docking experiments are employed to ascertain and evaluate the shape and orientation of ligands within their targets' binding sites. Conformations generated by search algorithms are evaluated according to their scoring functions (Torres et al. 2019). Moreover, the biological activities as well as affinities of leads toward specific targets proteins are predicted by molecular docking (Azmi et al. 2021). This study conducted an in silico analysis to visualize and evaluate the docking scores of BBR, CAF, and MTF against PD-related targets, including α-syn, TH, and PP2A. The number of H-bonds is closely related to the complex binding mode efficiency, which represents the greatest non-covalent intermolecular interactions (Shivanika et al. 2020). Above all, BBR exhibits a comparatively favorable interaction with α-syn Ser129 concerning CAF and MTF. Furthermore, the strong binding affinities of BBR and CAF, supported by their varied interactions with PLK-, CK2-, and GRK-mediated phosphorylation of α-syn active sites, underscore their potential as effective inhibitors for obstructing enzymes-mediated phosphorylation of α-syn. These in silico results elucidated the biological therapeutic properties of BBR and CAF as anti-PD agents, highlighting the binding scores, affinities, and intermolecular interactions between BBR or CAF and specific targets involved in the pathogenesis of PD. Further experimental studies are required to validate these predicted computational findings.

## Conclusions

In summary, the results of this investigation indicate that pre-treatment with a combination of BBR and CAF had a significant and promising neuroprotective effect against PD through their antioxidant and anti-inflammatory activities. This treatment can regulate inflammatory mediators like TNF-α and IL-6 and restore oxidative stress and antioxidant enzyme balance in ROT-induced rats. The combination of BBR and CAF significantly elevated PP2A levels, resulting in marked reduction of α-syn accumulation as well as motor deficits in ROT-induced mice. This combination increases dopamine levels and positively modulates TH enzyme activity, hence inhibiting ROT-induced alterations of dopaminergic neurotransmission, thereby contributing to their neuroprotective effects. Furthermore, through in silico study, we have suggested that BBR and CAF may be useful promising therapeutic agents for targeting the pathogenic processes associated with Parkinson's disease.

The outcomes of clinical trials are required to validate the benefits that were demonstrated in preclinical models. Experiments must be methodically designed to yield useful, reliable, and meaningful results. This planning must include quality control, study design, ethical, and financial considerations. The future goals of this research are to determine the ideal dosages, evaluate the safety over the long term, and investigate the possibility of incorporating BBR and CAF into the conventional therapy protocols for Parkinson's patients. The combination of BBR and CAF has significant promise to delay the onset of Parkinson's disease and mitigate its progression.

## Electronic supplementary material

Below is the link to the electronic supplementary material.

Supplementary material 1 (DOC 7647 kb)

## Data Availability

All data generated or analyzed during this study are included in this article and supplementary file.
