# Supplementary material for: Neuroprotective efficacy of berberine and caffeine against rotenone‐induced neuroinflammatory and oxidative disturbances associated with Parkinson’s disease via inhibiting α-synuclein aggregation and boosting dopamine release
Source: Inflammopharmacology. 2025 Mar 9;33(4):2129–50. doi: 10.1007/s10787-025-01661-w (PMC11991993; doi:10.1007/s10787-025-01661-w)
Supplement: Supplementary file 1 — Supplementary material 1 (DOC 7647 kb) [file 10787_2025_1661_MOESM1_ESM.docx]

**Table S1 Molecular docking results.**

| **compound** | **Affinity (kcal/mol)** | **CNN affinity** | **CNN pose score** |
| --- | --- | --- | --- |
| **Alpha-synuclein (α-syn Ser129)** | | | |
| Berberine (BBR) | -5.11 | 4.31 | 0.53 |
| Caffeine (CAF) | -3.26 | 3.07 | 0.72 |
| Metformin (MTF) | -3.23 | 2.69 | 0.68 |
| **Polo-like kinase (PLK)** | | | |
| BBR | -8.49 | 6.34 | 0.30 |
| CAF | -5.90 | 4.96 | 0.93 |
| MTF | -3.87 | 3.61 | 0.61 |
| co-crystal ligand | -8.58 | 7.36 | 0.97 |
| **Casein kinase 2 (CK2)** | | | |
| BBR | -9.09 | 6.84 | 0.77 |
| CAF | -5.78 | 5.26 | 0.95 |
| MTF | -4.29 | 3.84 | 0.63 |
| co-crystal ligand | -10.19 | 8.10 | 0.99 |
| **G protein-coupled receptor kinase (GRK)** | | | |
| BBR | -11.06 | 6.91 | 0.70 |
| CAF | -6.44 | 5.12 | 0.78 |
| MTF | -5.02 | 3.35 | 0.37 |
| co-crystal ligand | -8.85 | 5.53 | 0.46 |

| 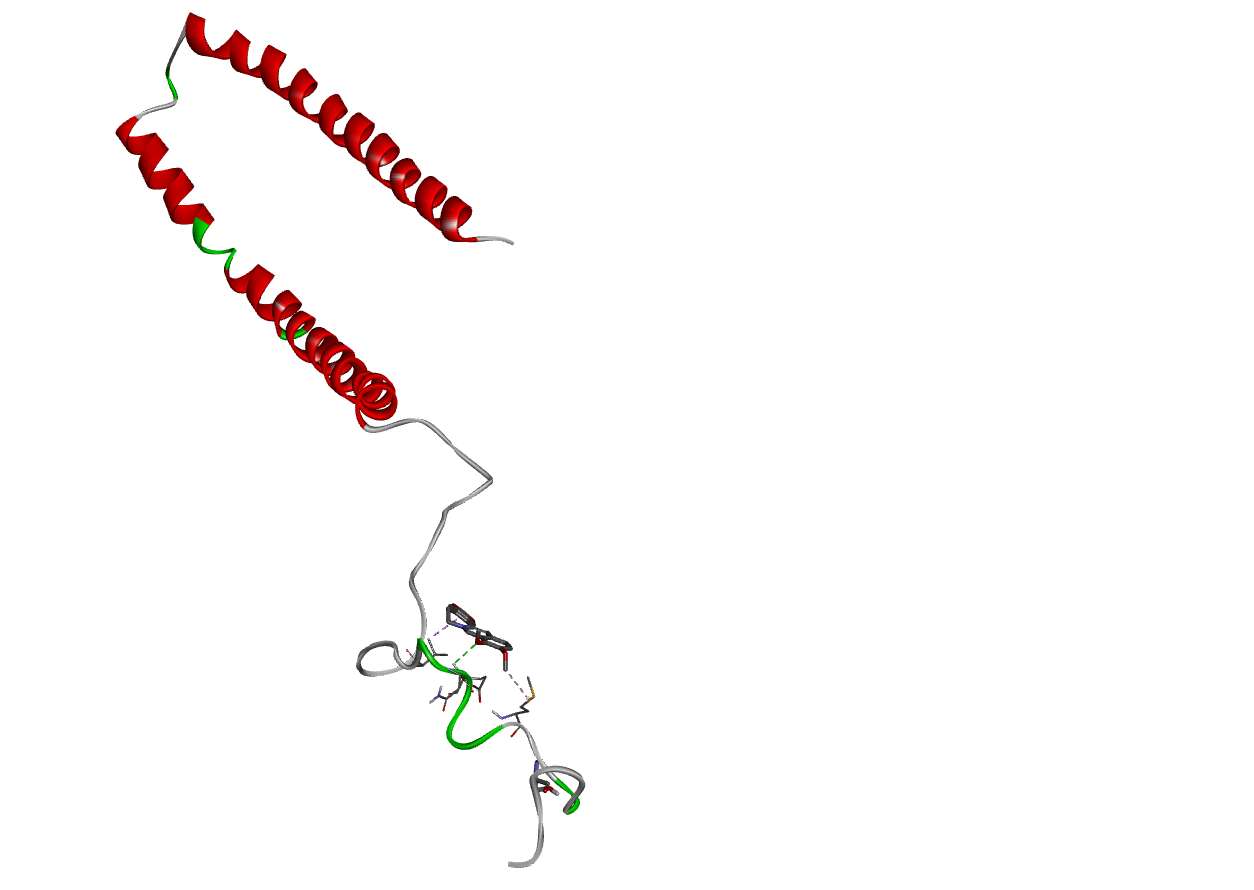 | | 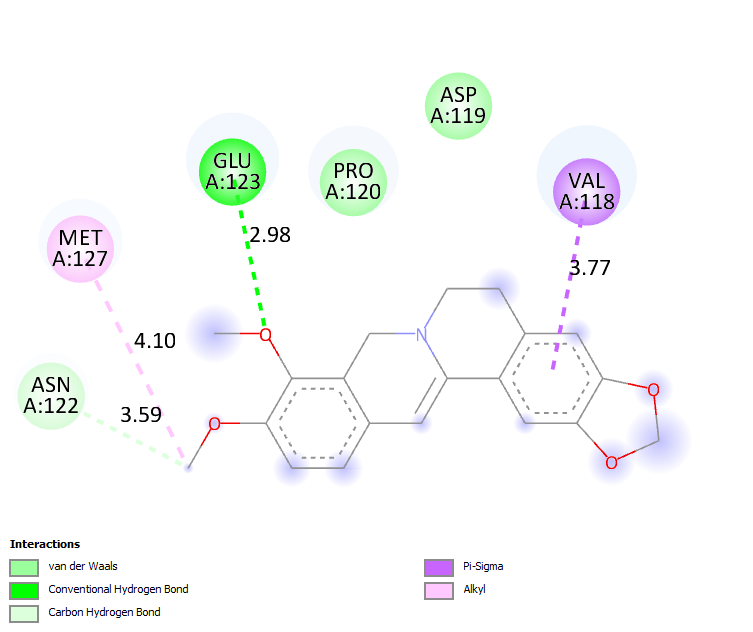 | |
| --- | --- | --- | --- |
| **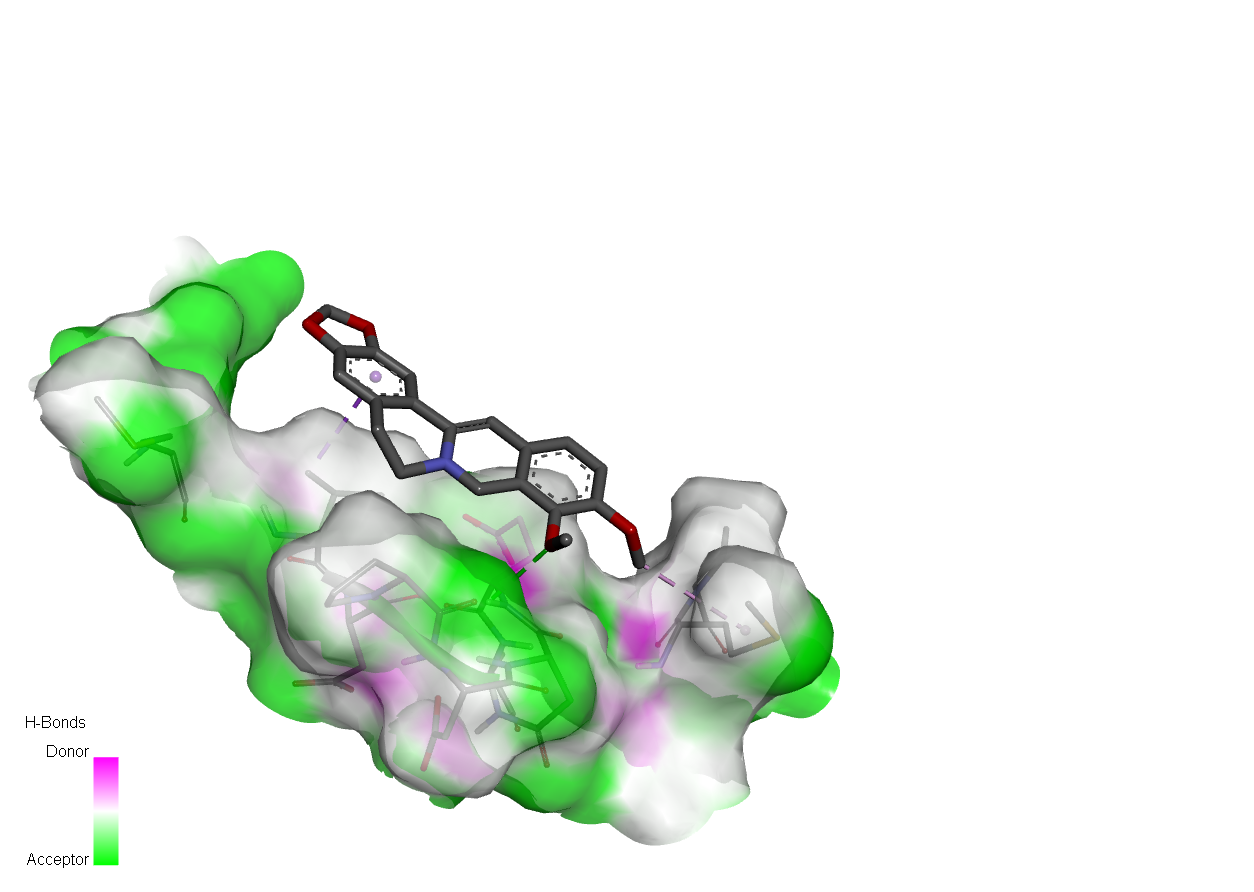** | |  | |
| **(A)** | | | |
| **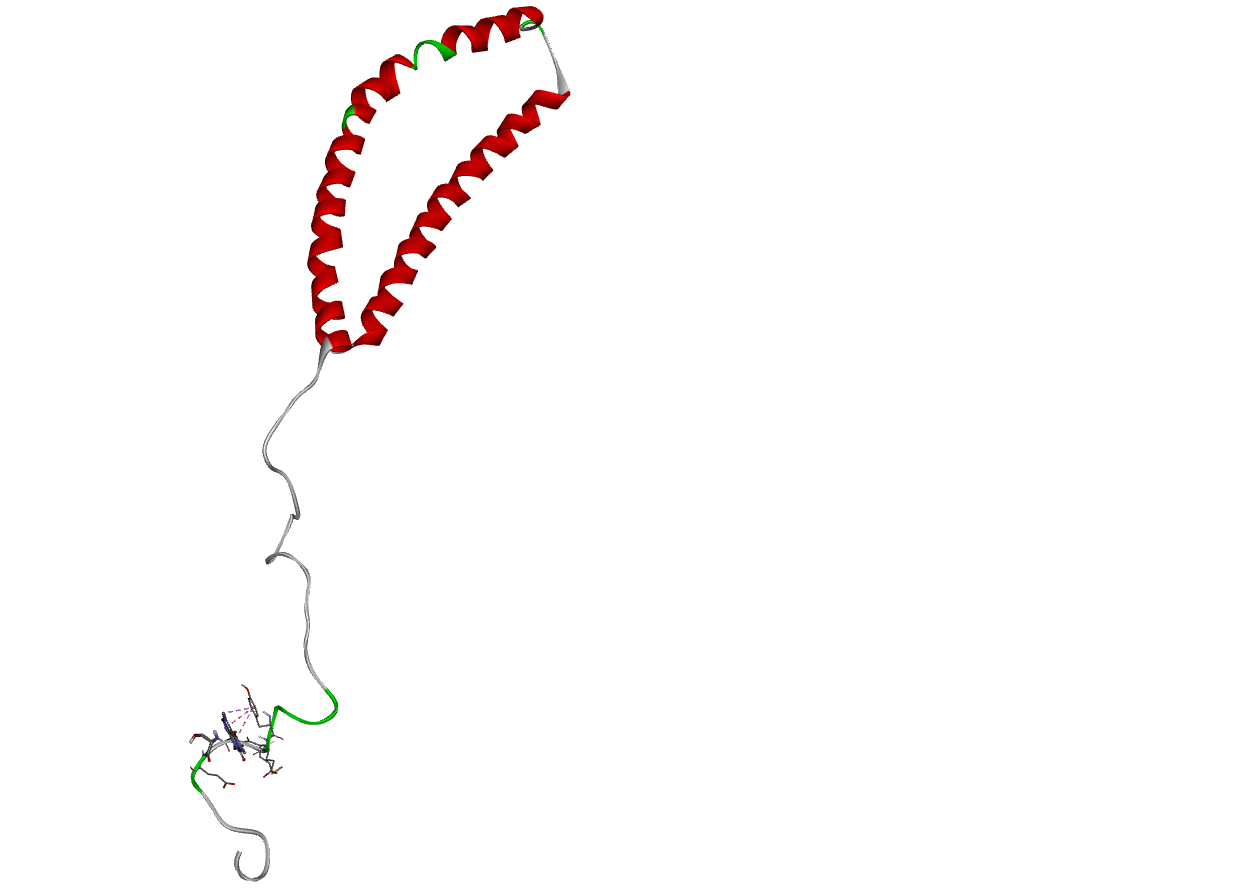** | | | **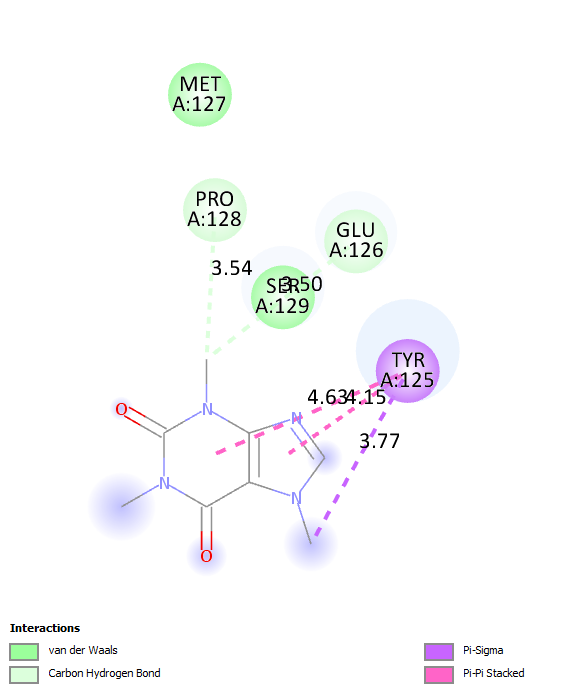** |
| **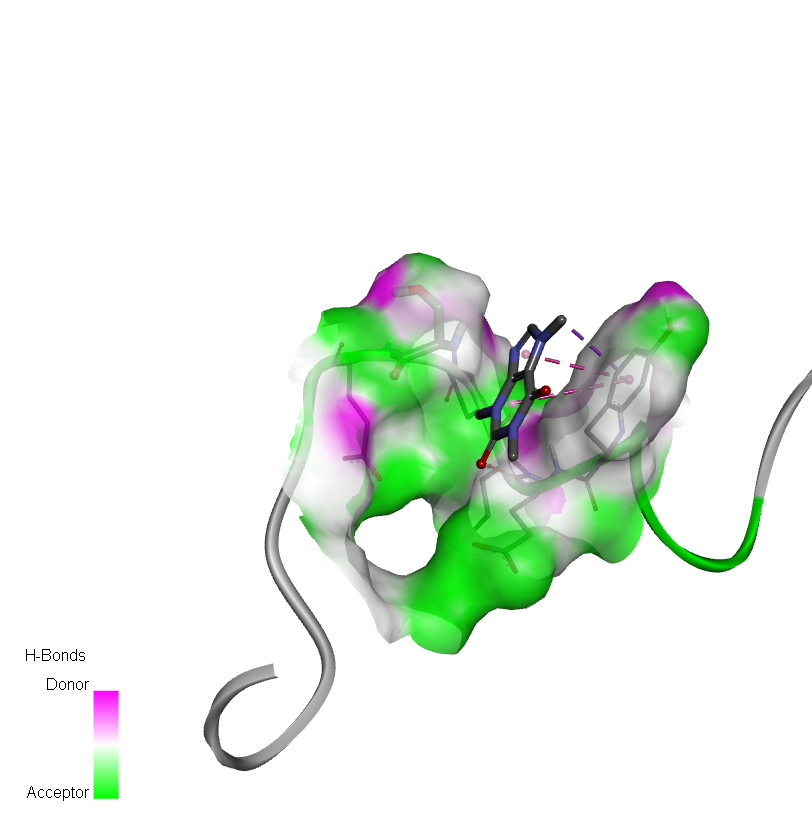** | | |  |
| **(B)** | | | |
| 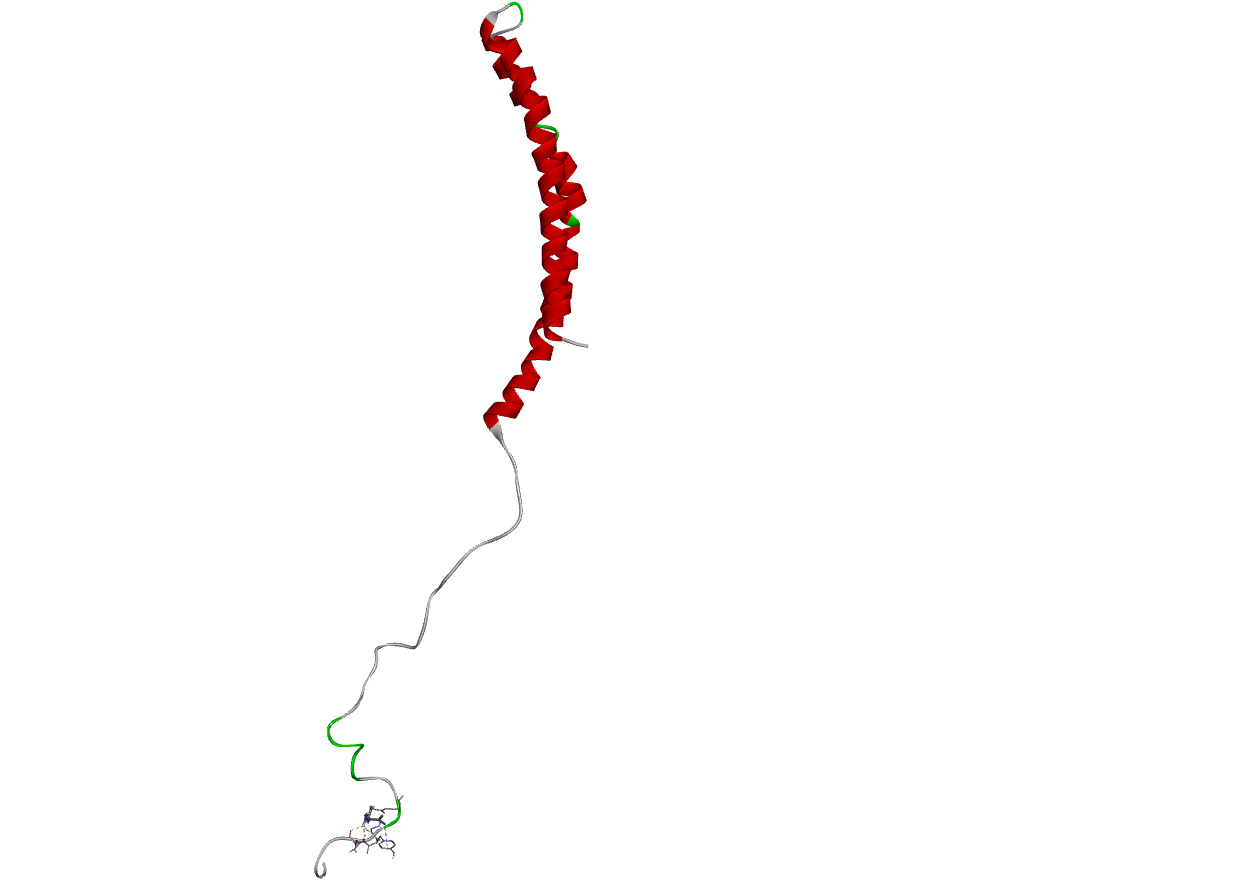 | 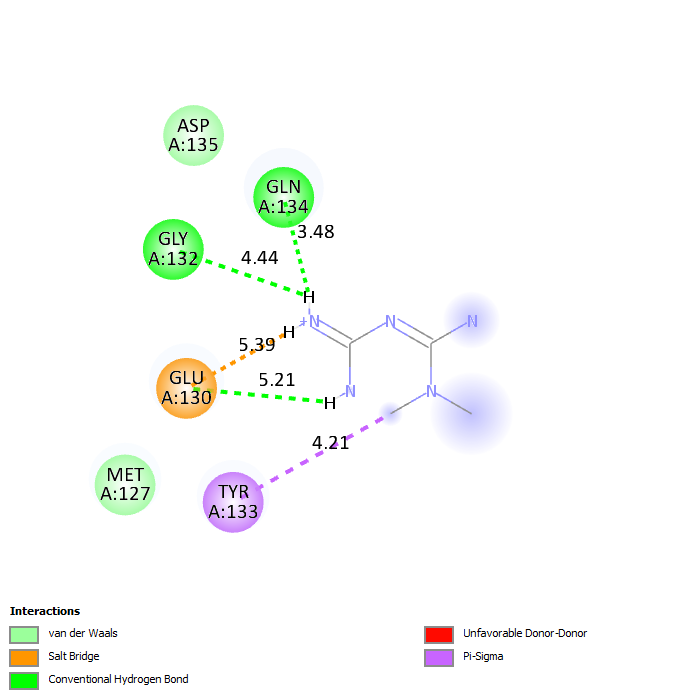 | | |
| **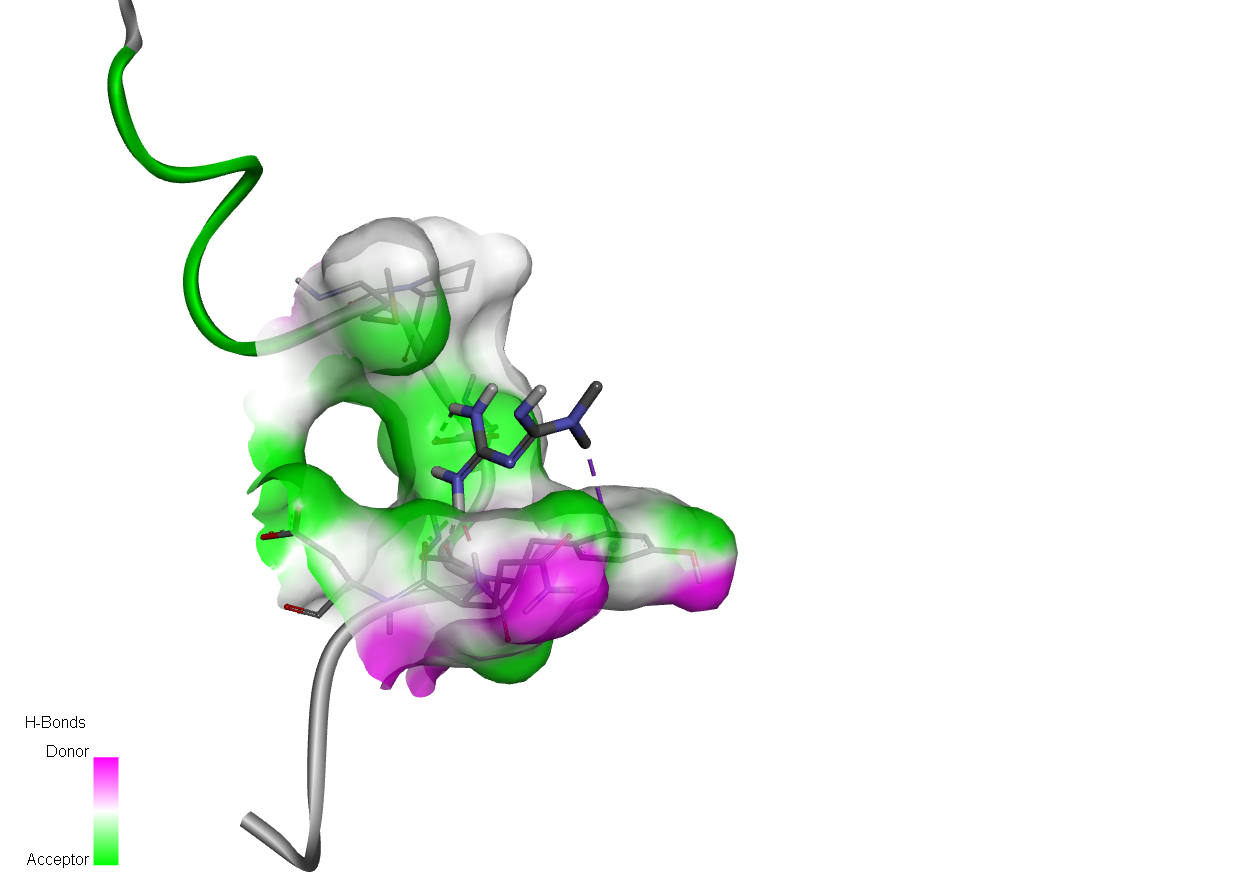** | | | |
| **(C)** | | | |

**Fig. 1 Docking analysis against α-syn Ser129.** (**A**) Docking of BBR with α-syn with the grid box being set at residue Ser129 and Blind docking of BBR with α-syn showing the generated nine poses (with affinities around -3.67 kcal/mol) none of them bound to Ser129 residue, (**B**) Docking of CAF with α-syn with the grid box being set at residue Ser129 and Blind docking of CAF with α-syn showing the generated nine poses (with affinities around -3.67 kcal/mol) none of them bound to Ser129 residue and (**C**) Docking of MTF with α-syn with the grid box being set at residue Ser129.

| **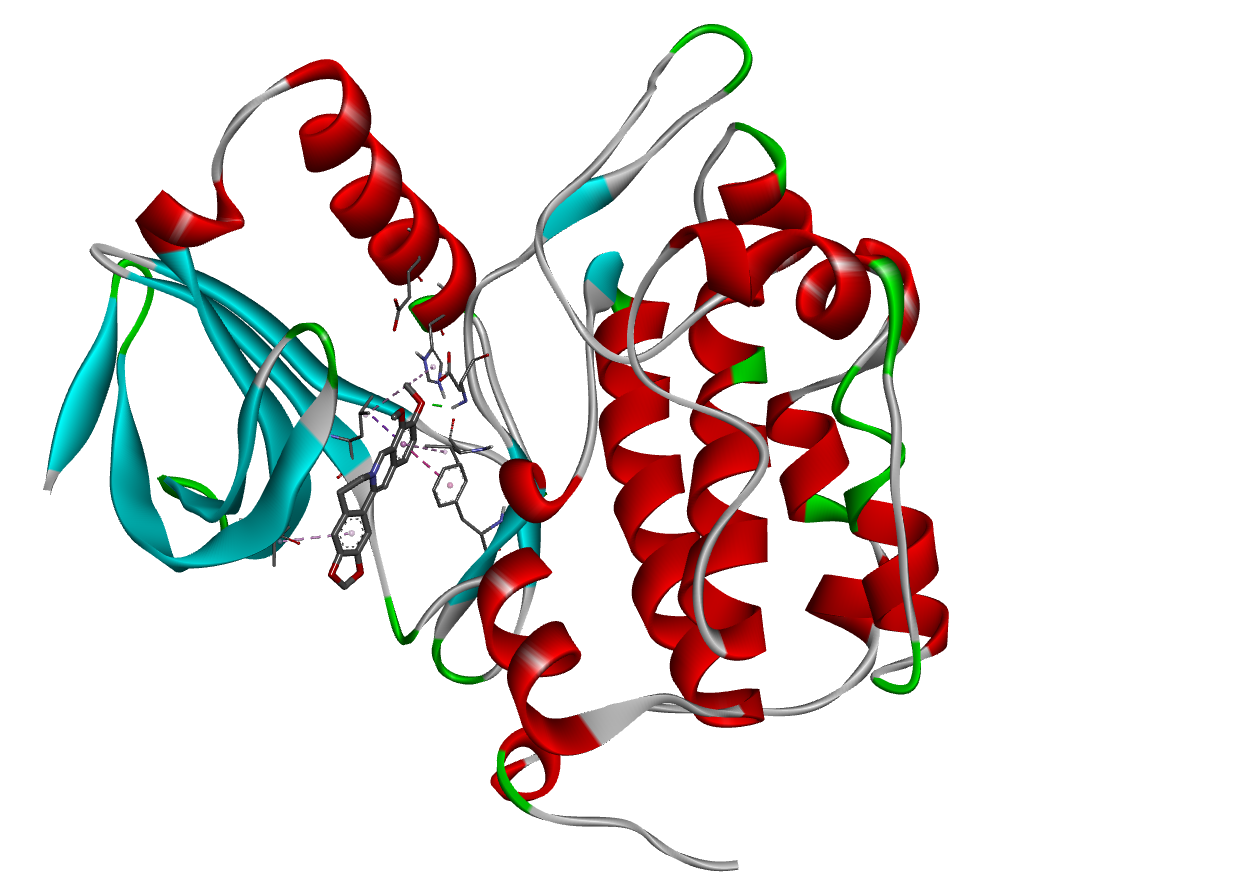** | **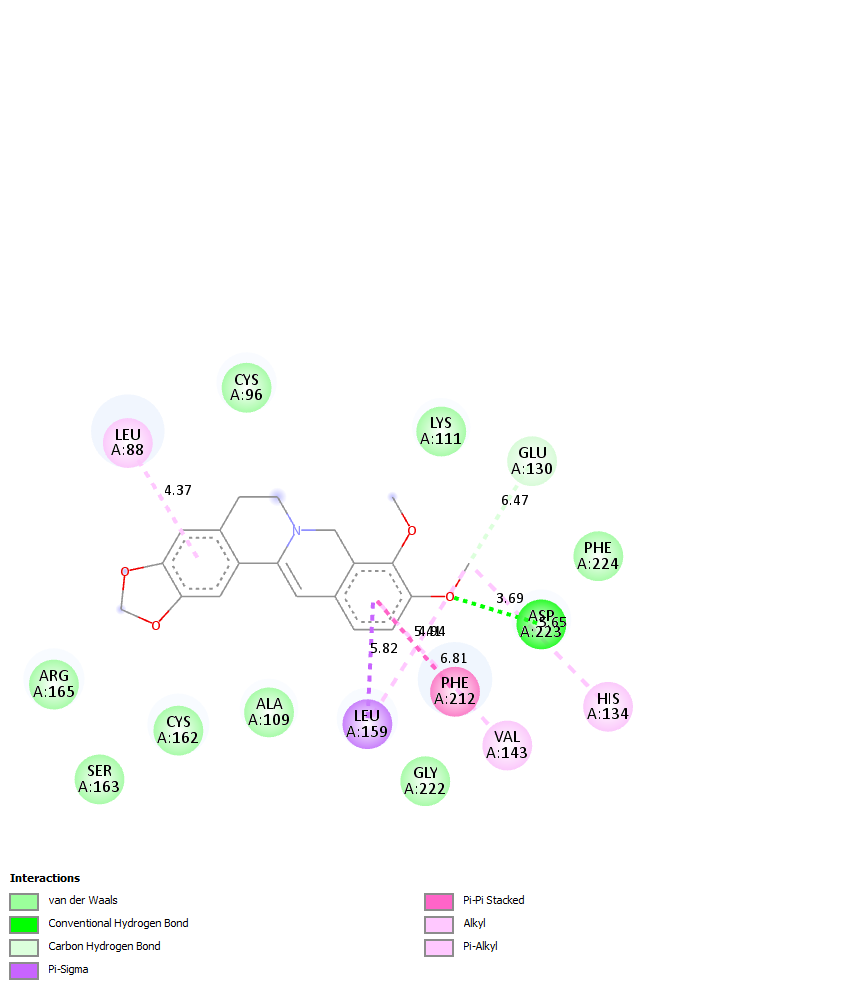** | | | |  |  |  |
| --- | --- | --- | --- | --- | --- | --- | --- |
| **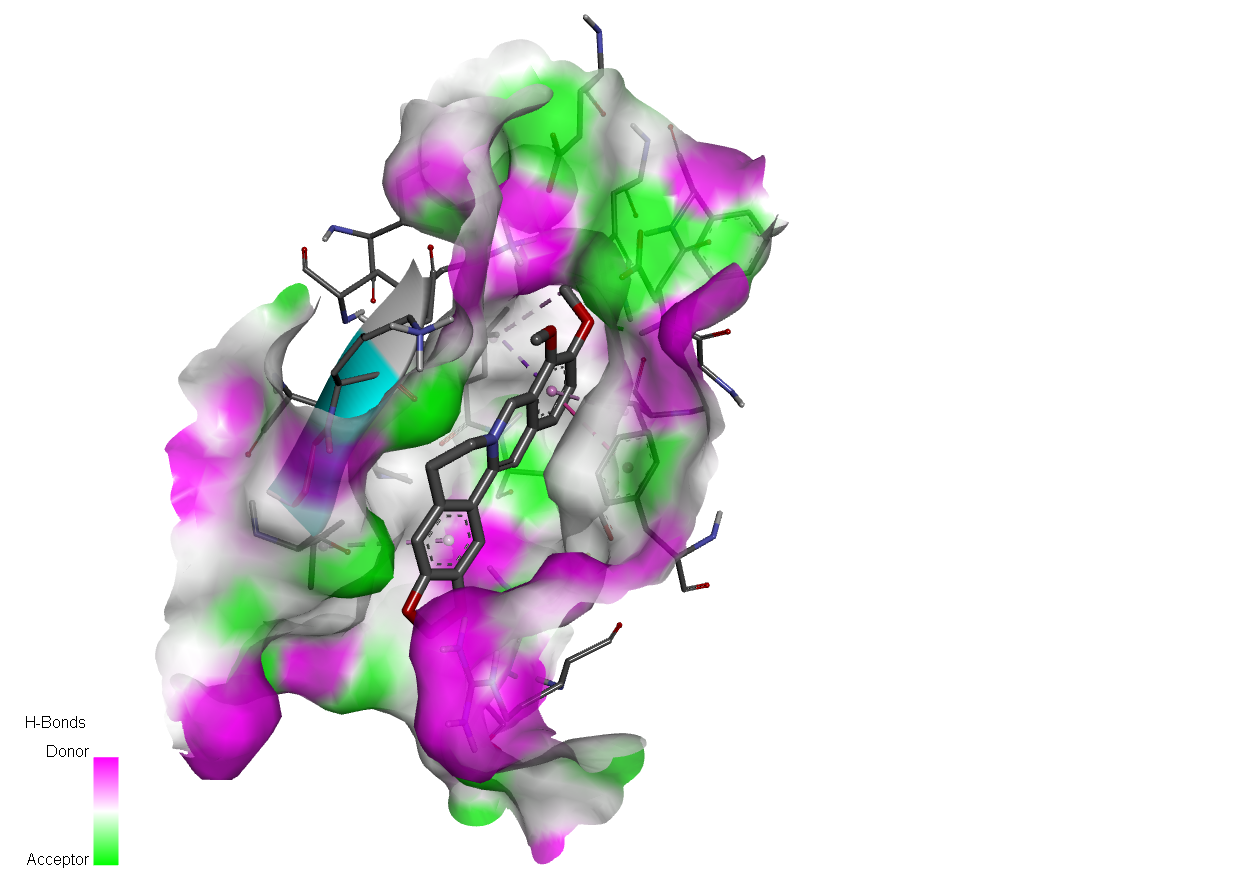** | | | | |  |  |  |
| **(A)** | | | | |  |  |  |
| **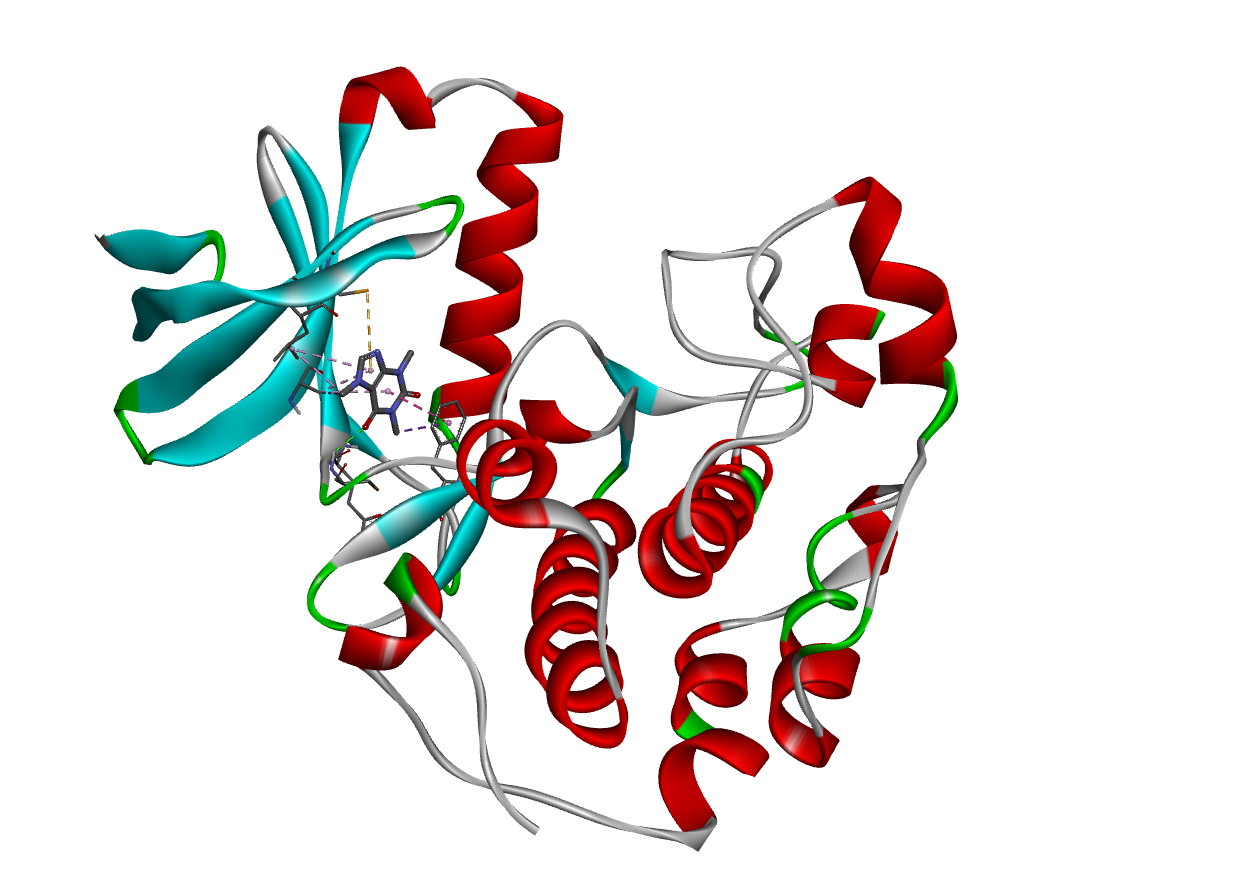** | | | | **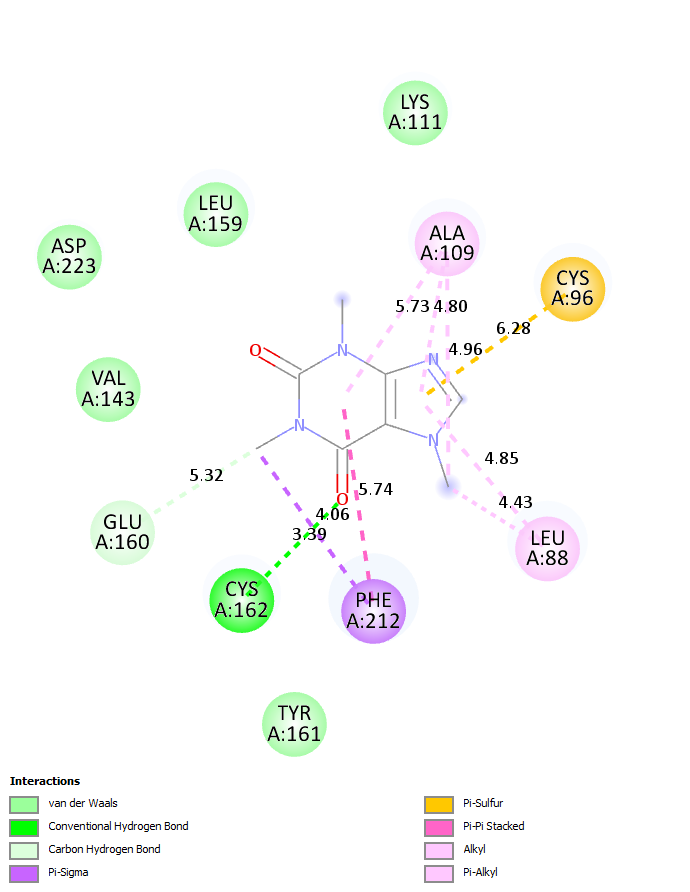** |  |  |  |
| **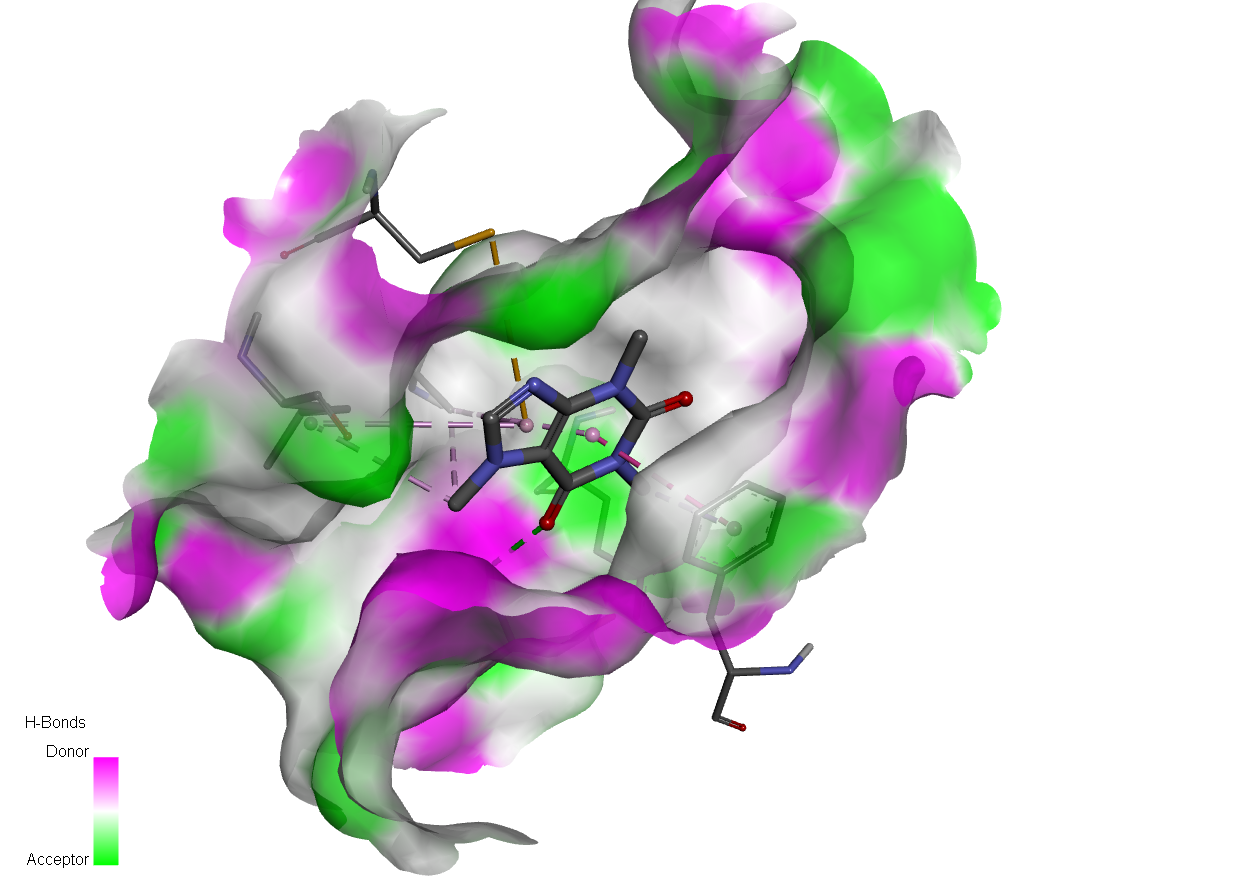** | | | | |  |  |  |
| **(B)** | | | | |  |  |  |
| **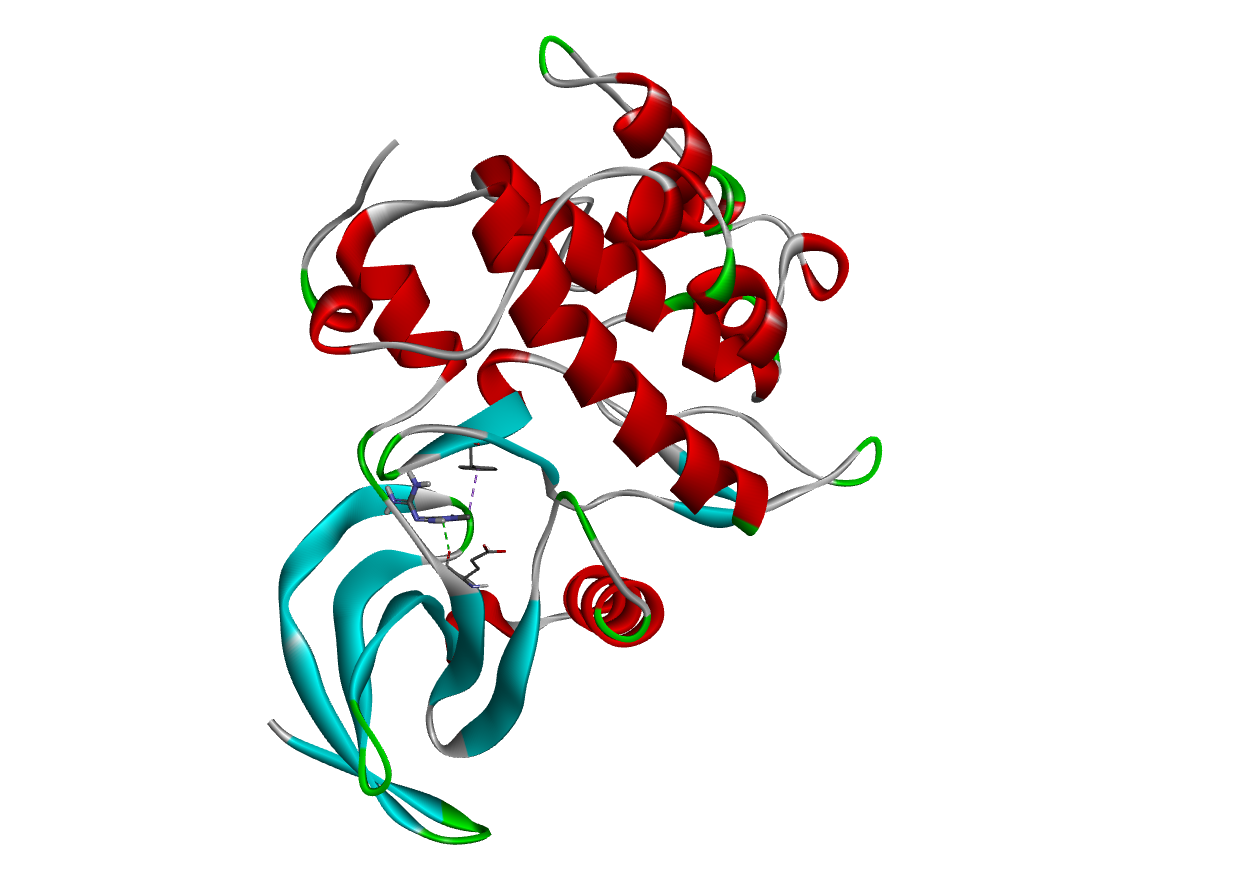** | | | | **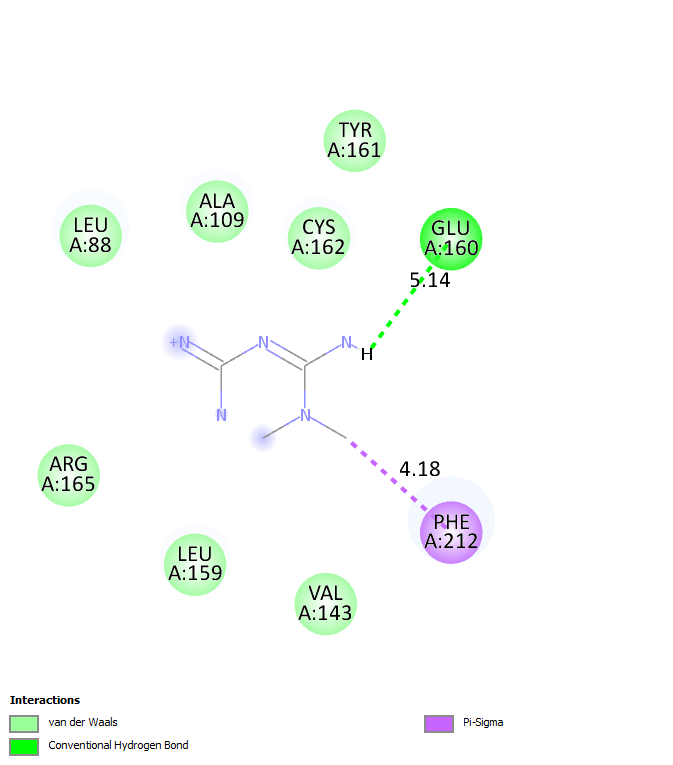** | | | |
| **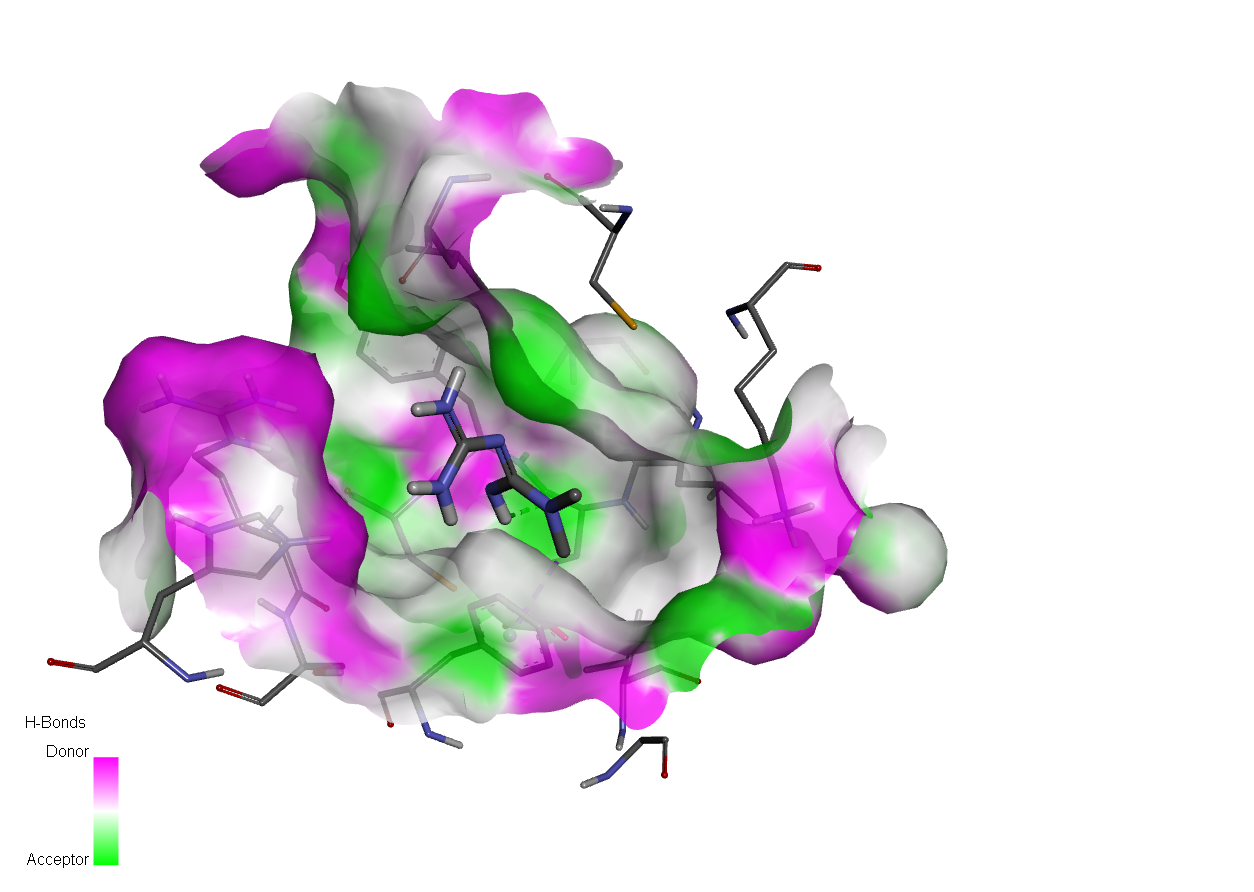** | | | | | | | |
| **(C)** | | | | | | | |

**Fig. 2 Docking analysis against PLK.** (**A**) Docking of BBR with PLK, (**B**) Docking of CAF with PLK and (**C**) Docking of MTF with PLK.

**
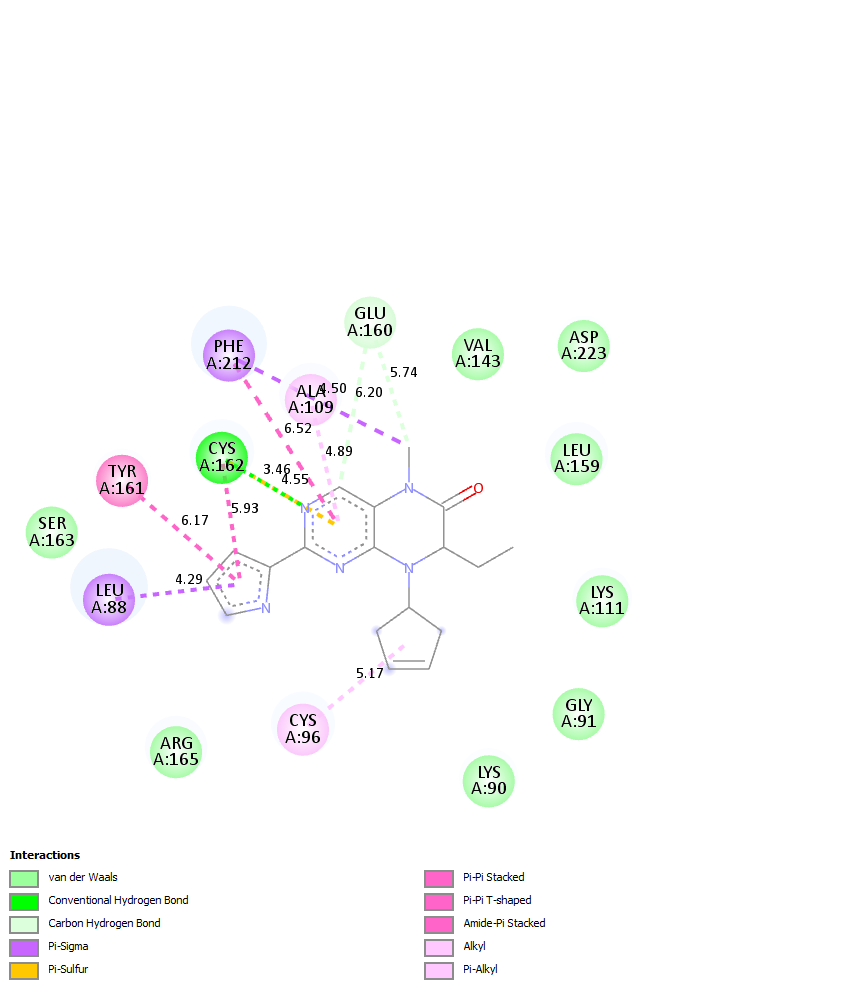
**

**Fig. 3 Docking of the co-crystal ligand with PLK.**

| **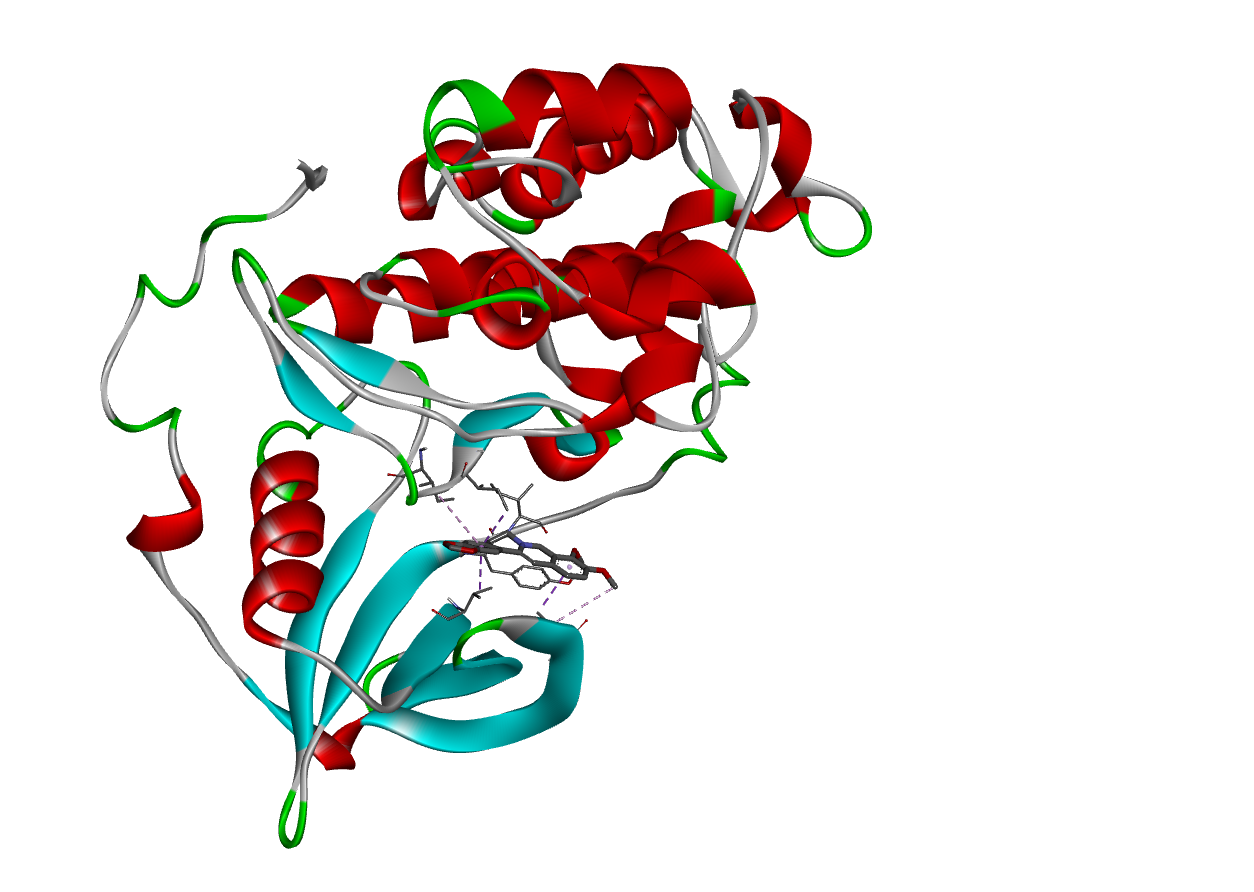** | | **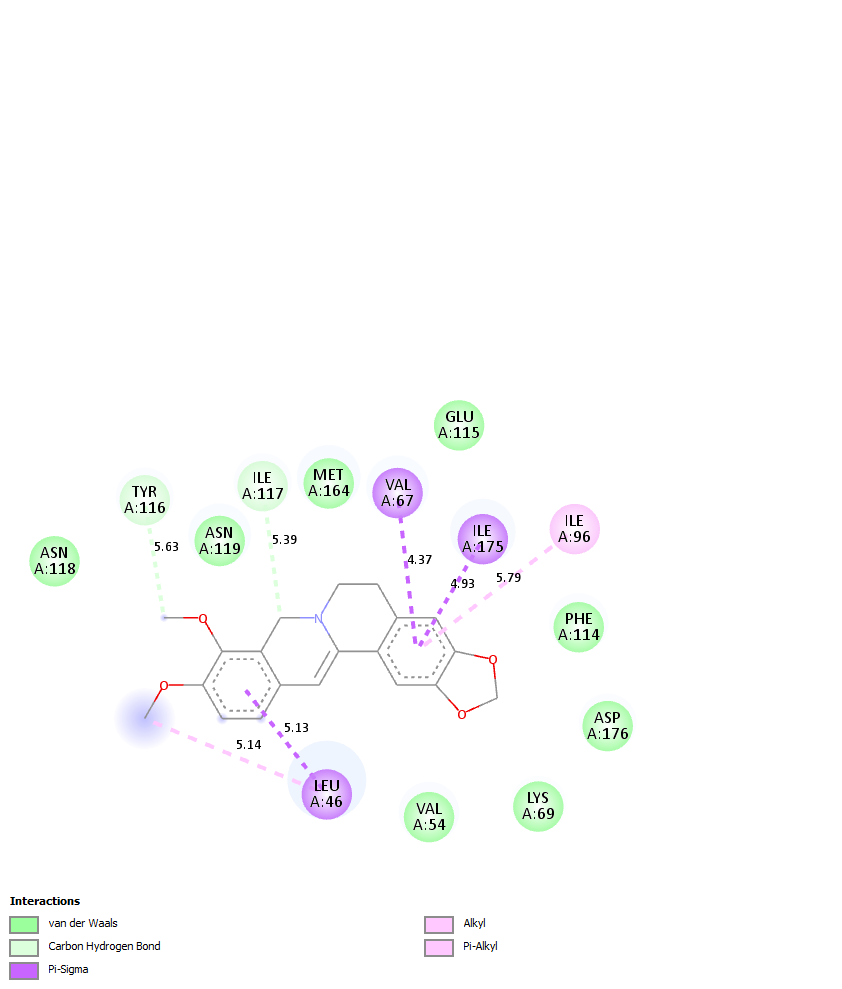** | |
| --- | --- | --- | --- |
| **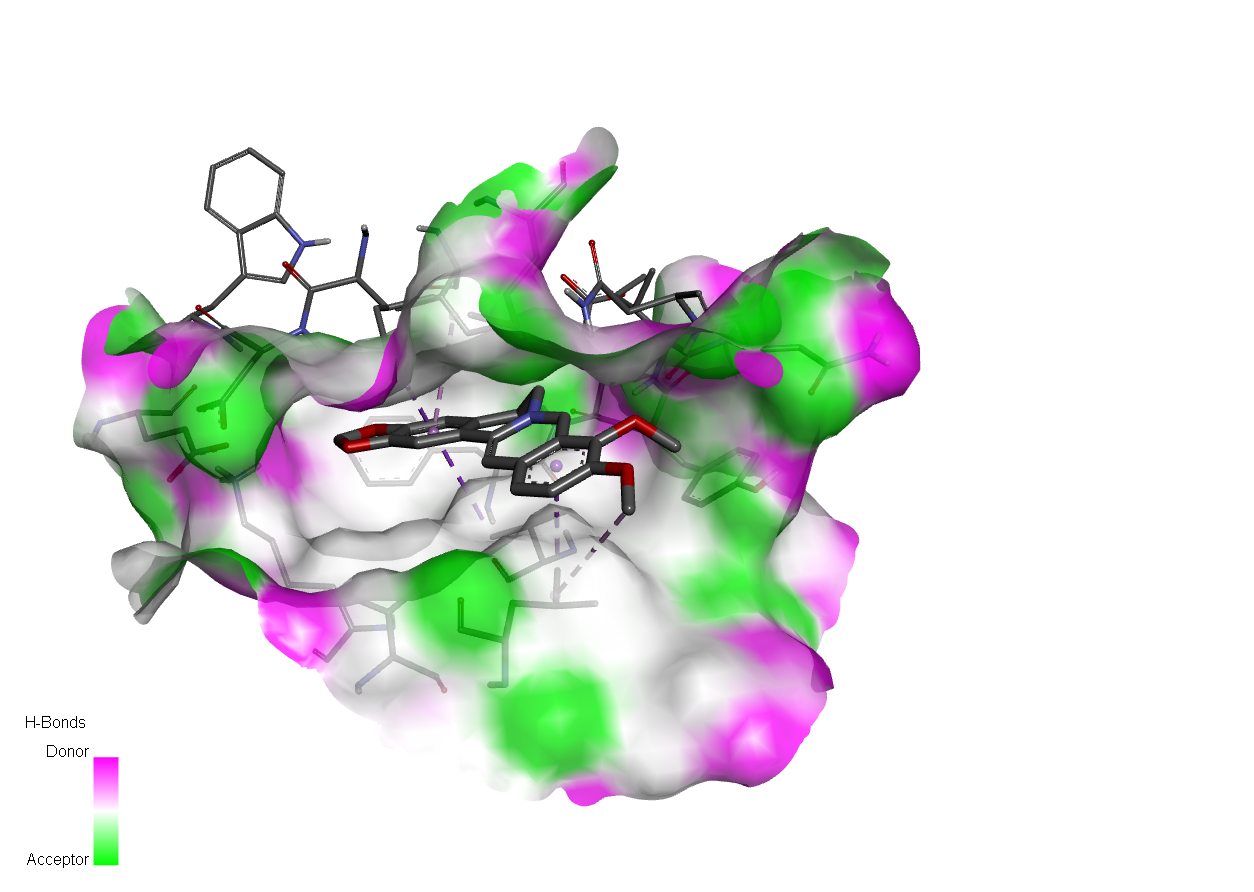** | | | |
| **(A)** | | | |
| **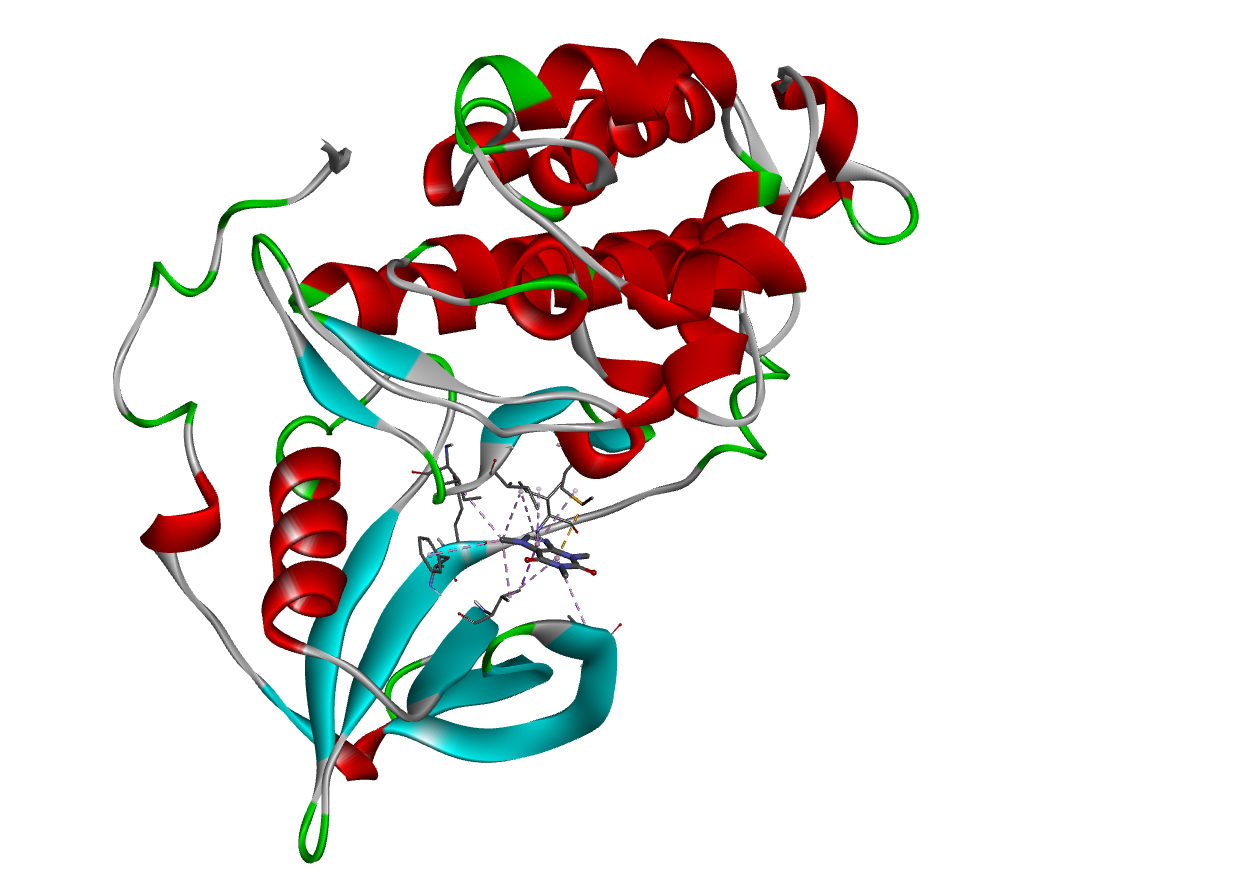** | | | **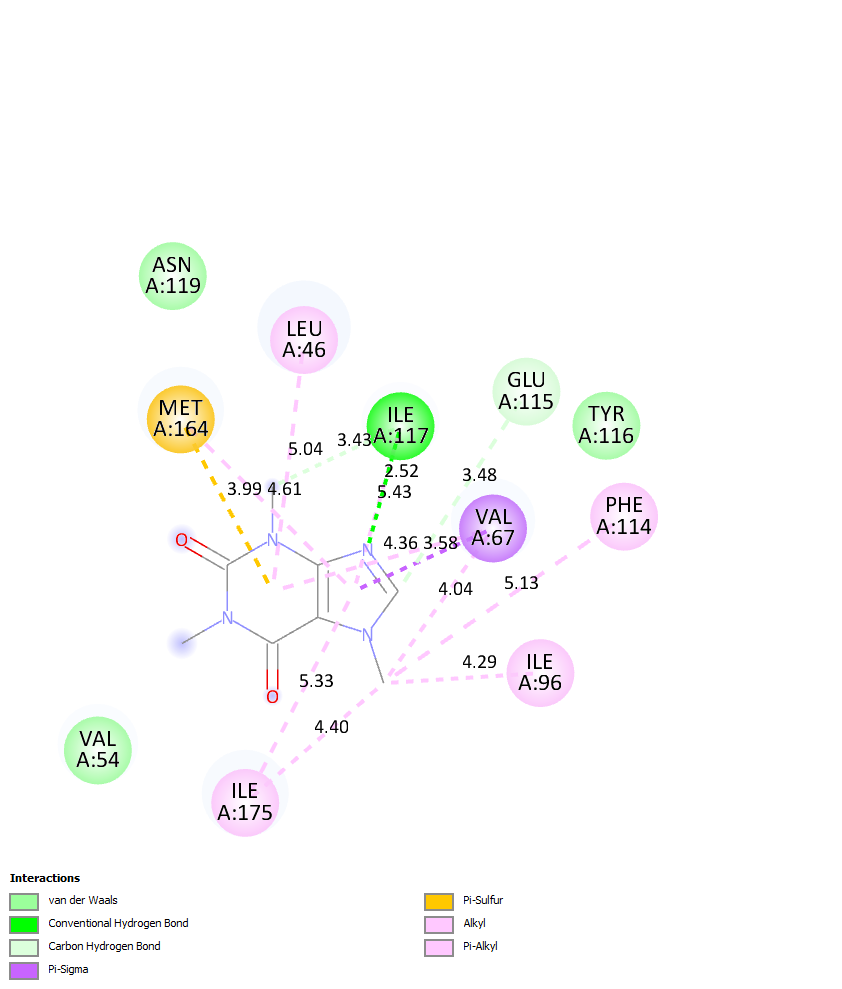** |
| **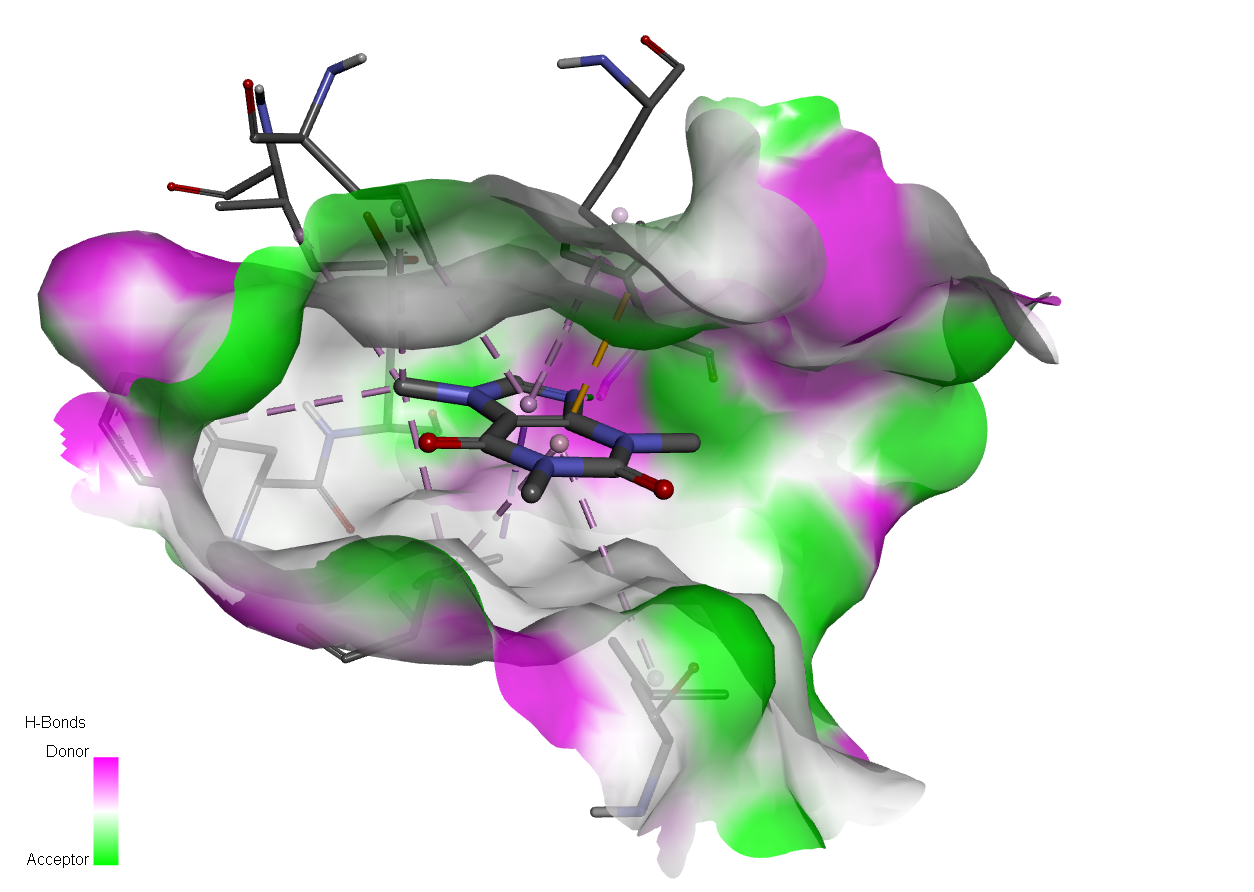** | | | |
| **(B)** | | | |
| **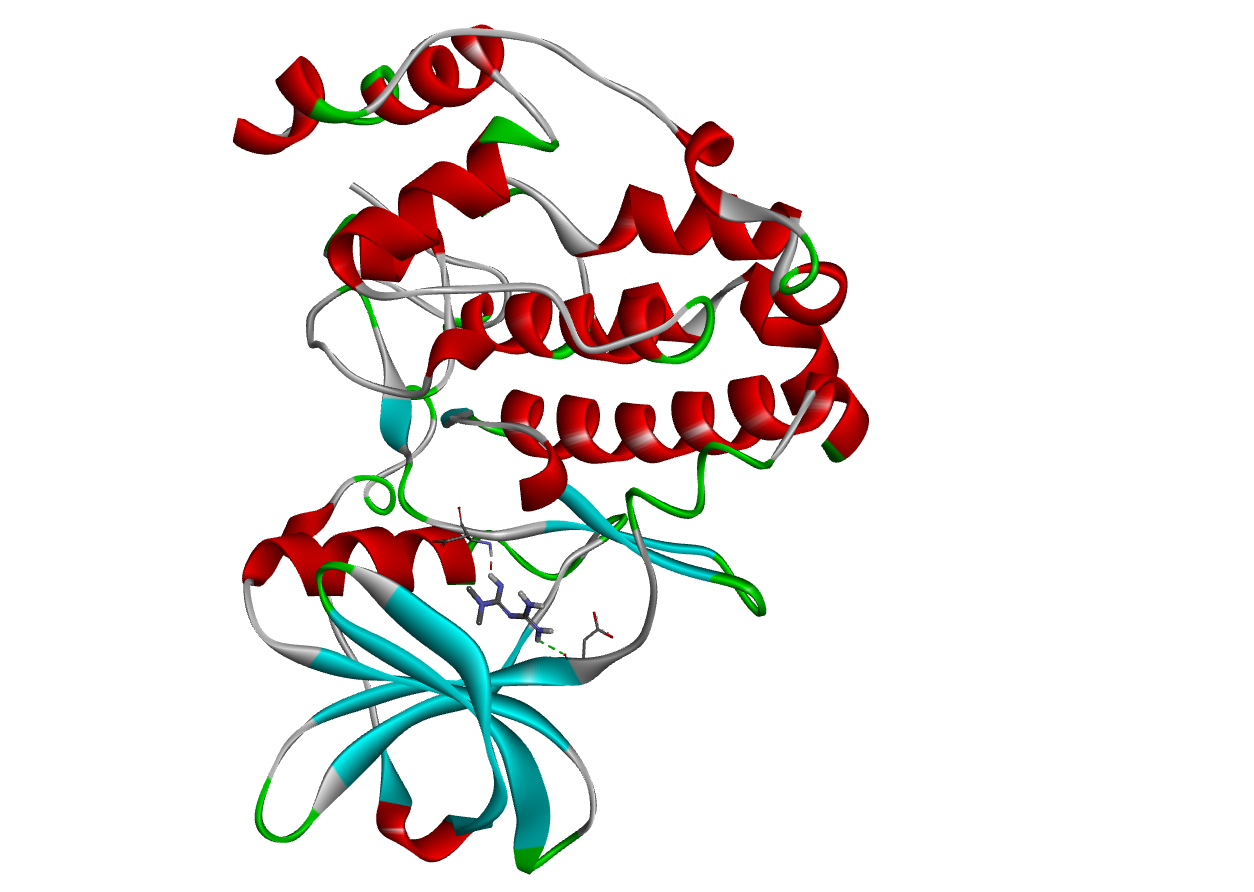** | **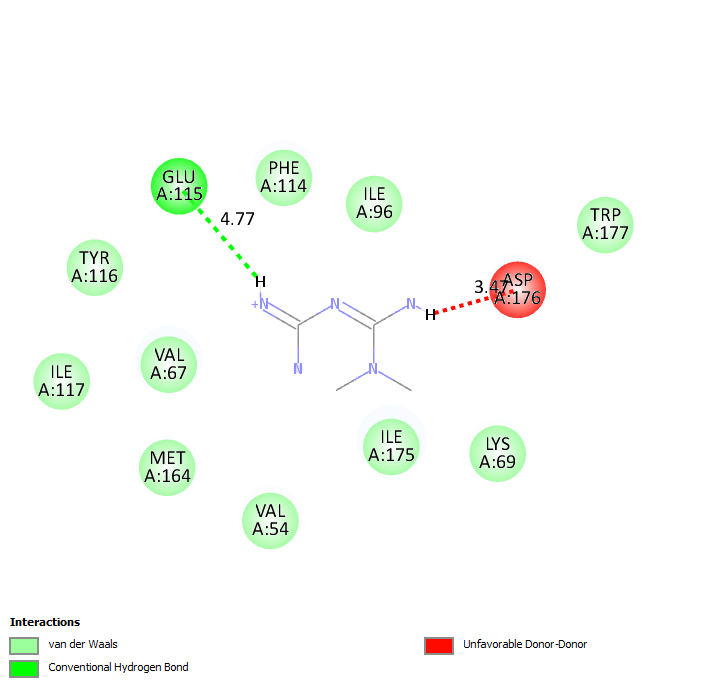** | | |
| **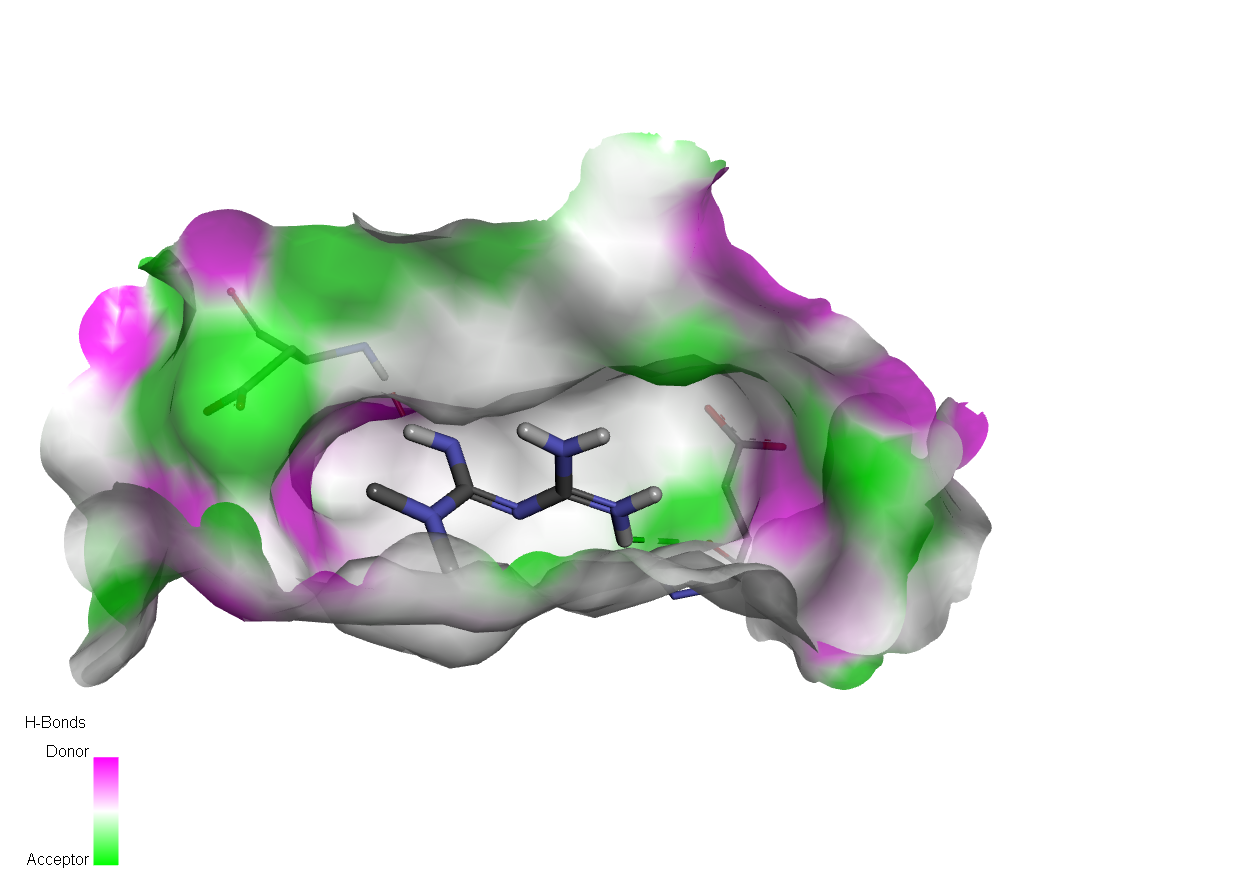** | | | |
| **(C)** | | | |

**Fig. 4 Docking analysis against CK2.** (**A**) Docking of BBR with CK2, (**B**) Docking of CAF with CK2 and (**C**) Docking of MTF with CK2.


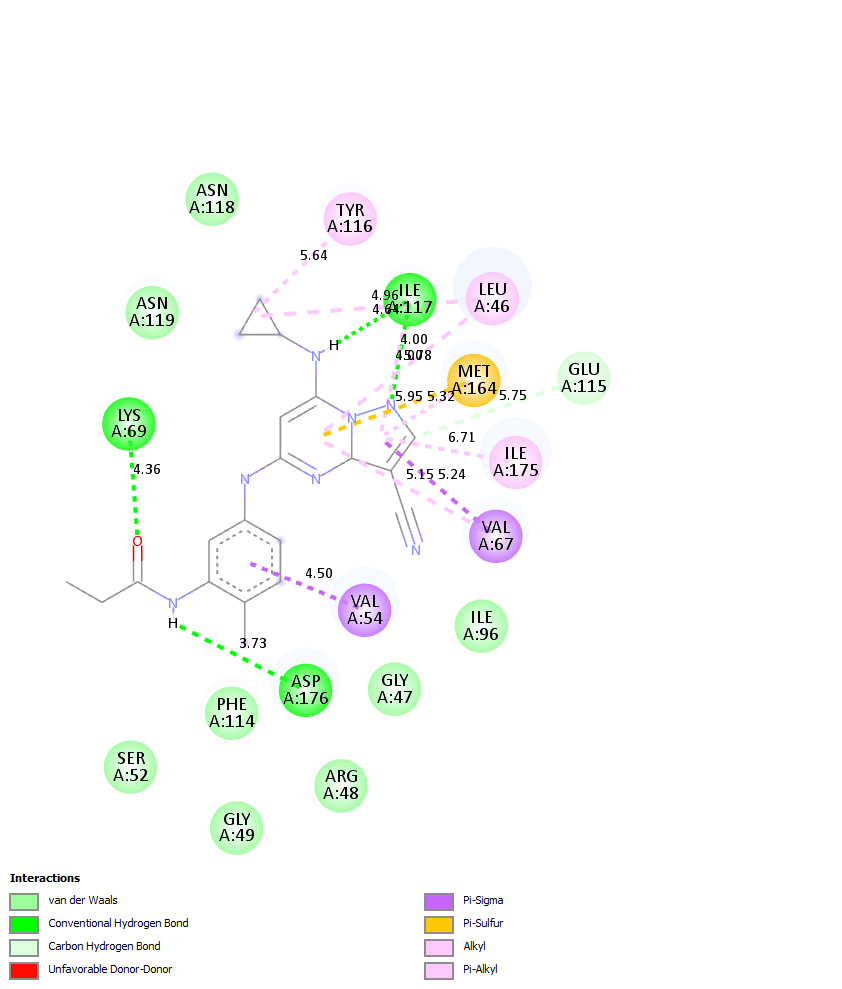


**Fig. 5 Docking of the co-crystal ligand with CK2**

| **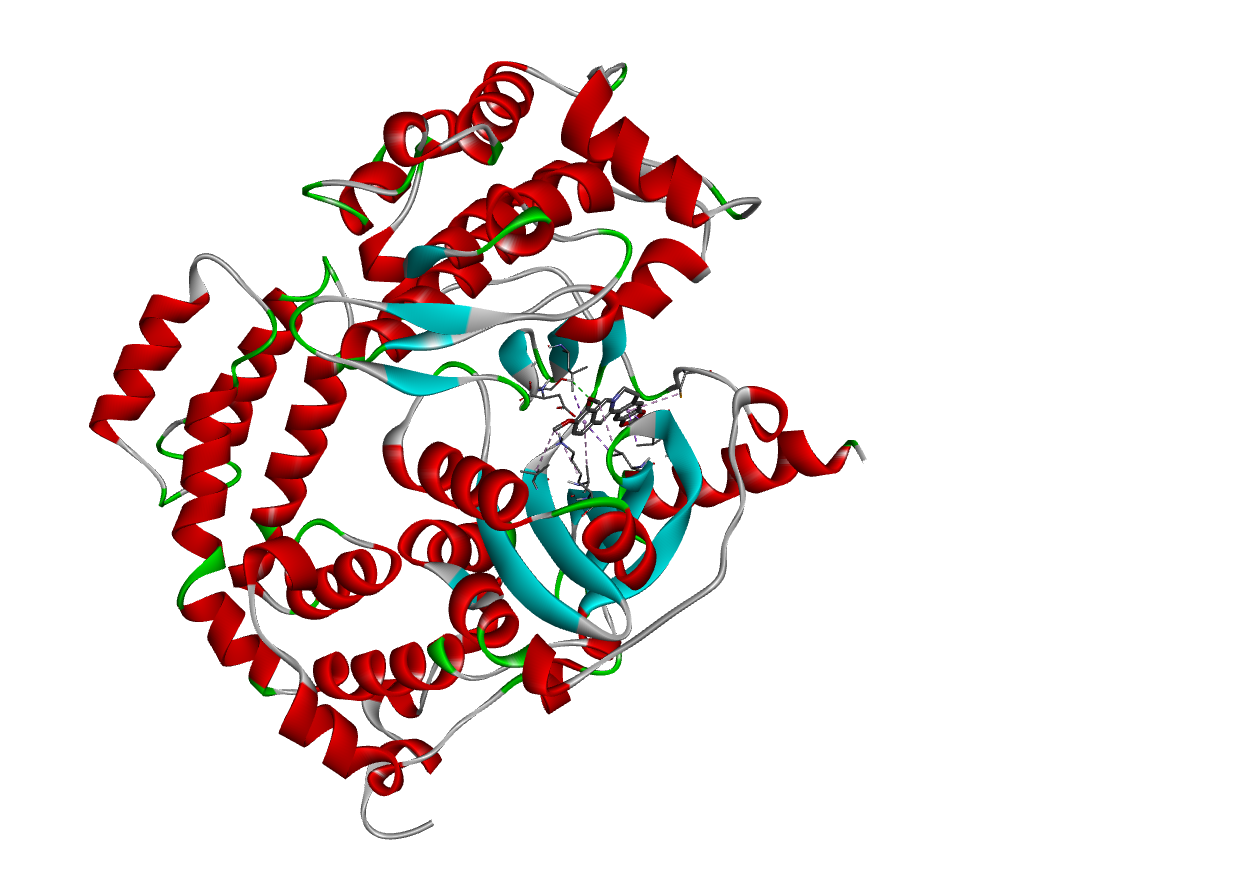** | | **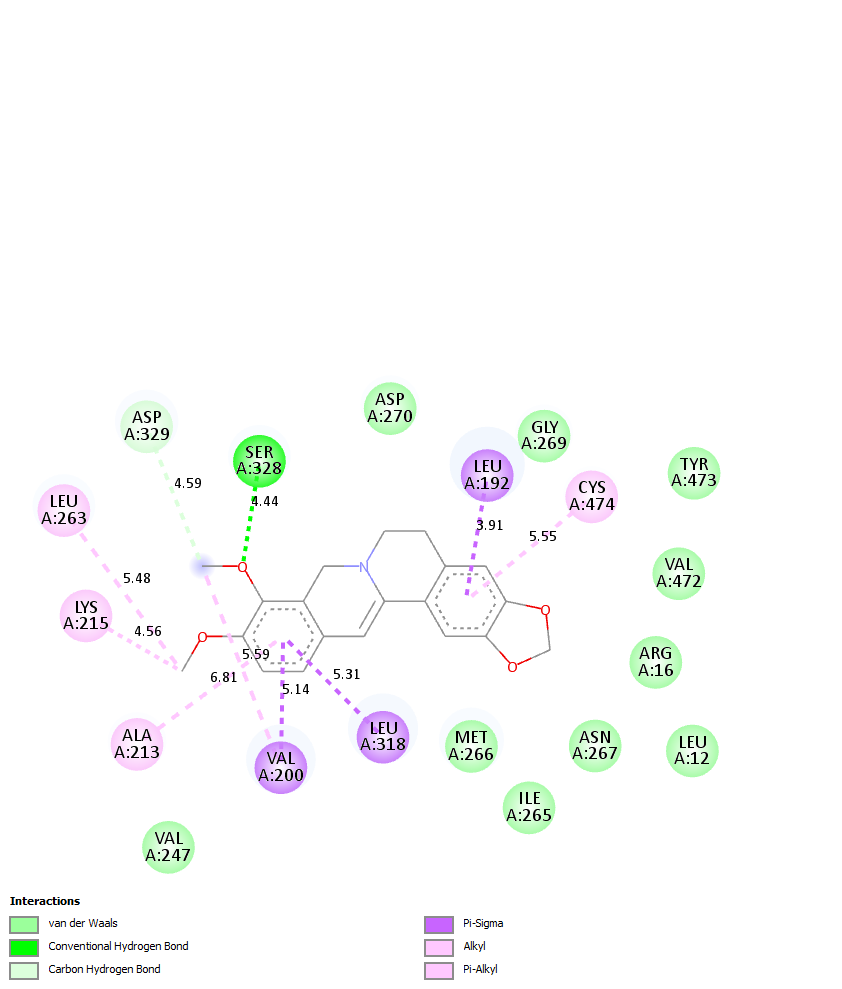** | | |
| --- | --- | --- | --- | --- |
| **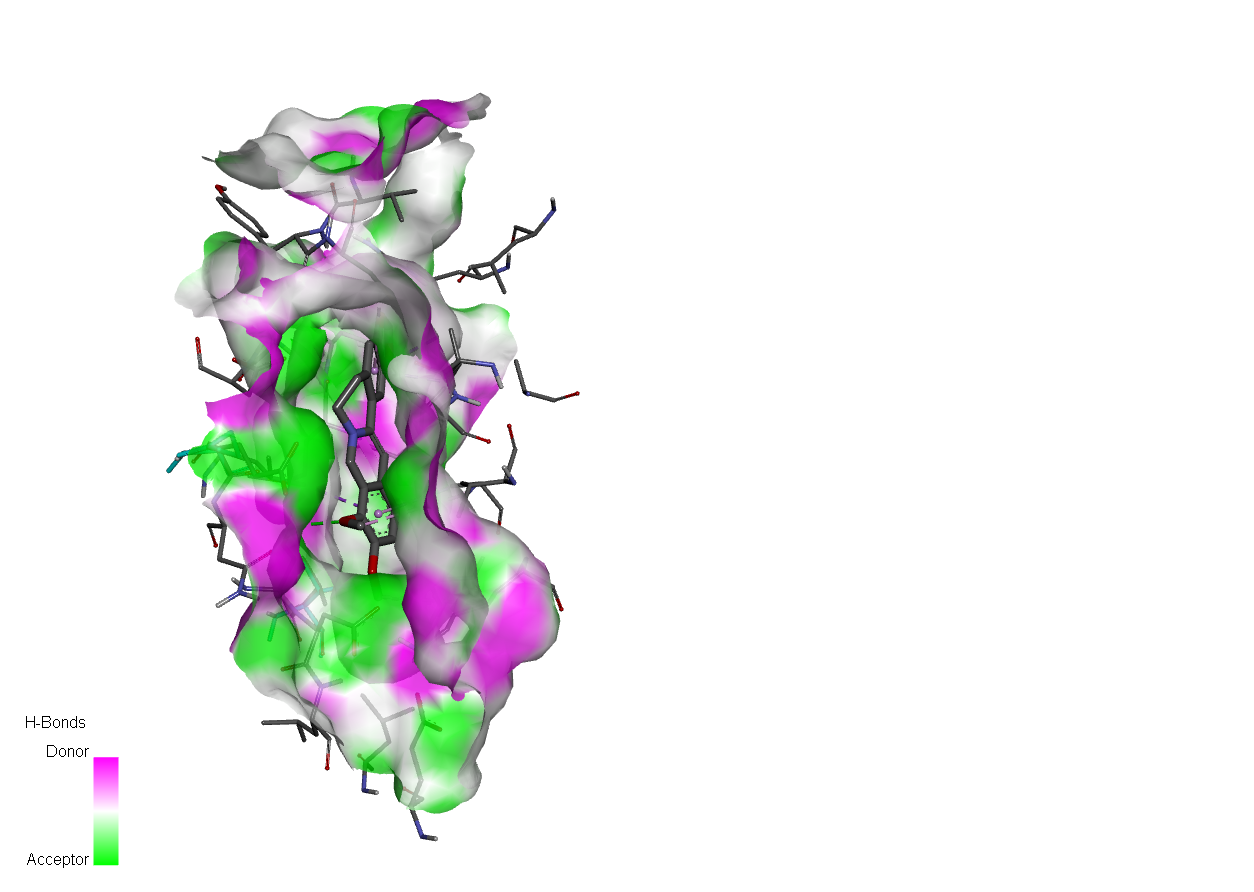** | | | | |
| **(A)** | | | | |
| **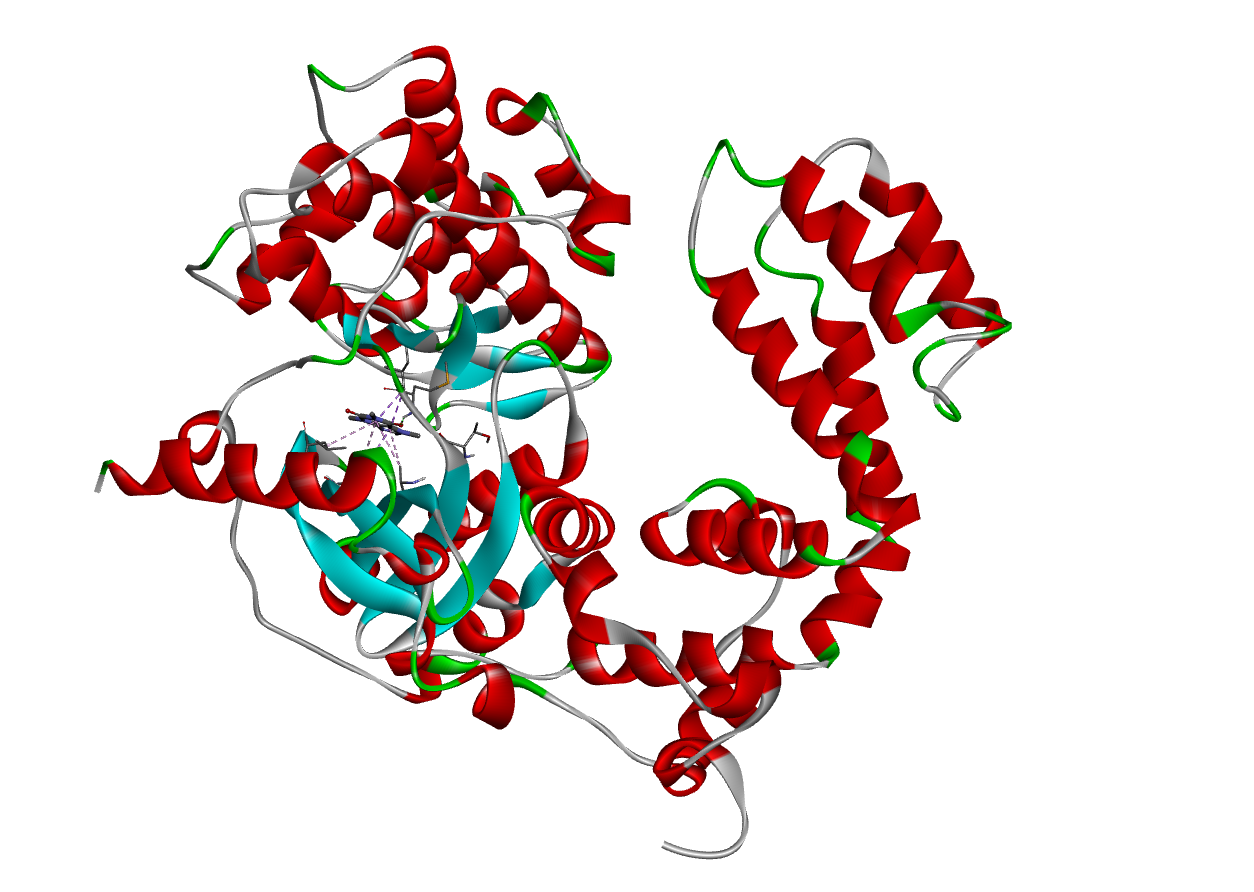** | | | | **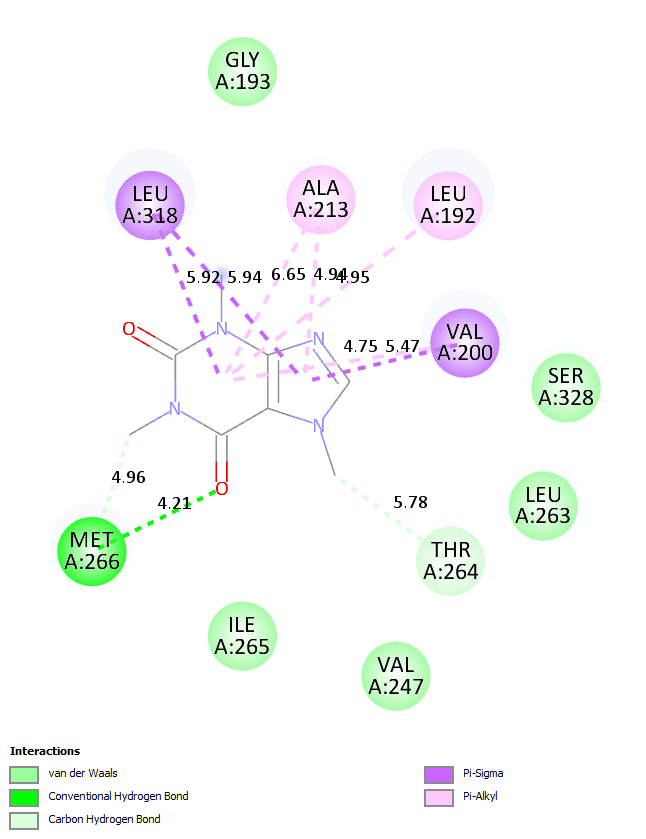** |
| **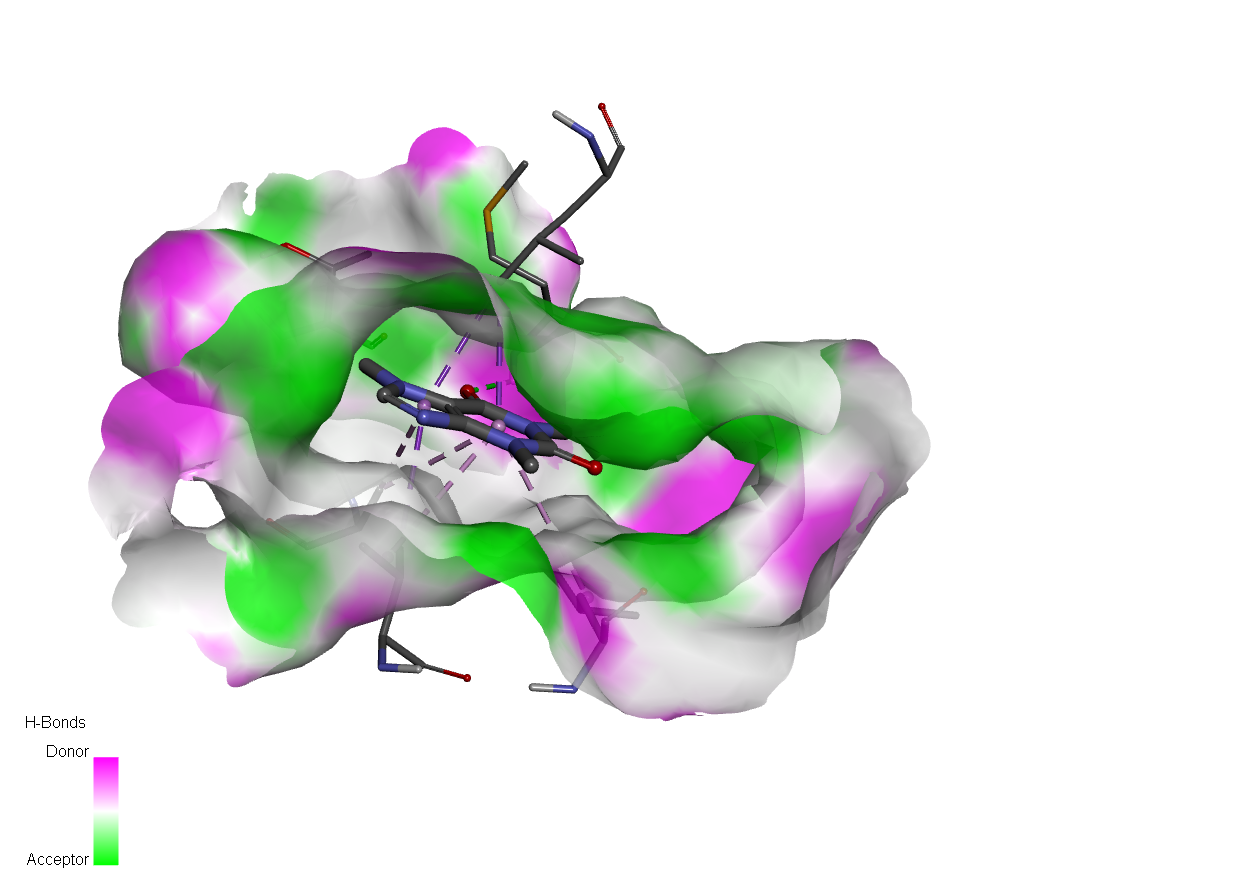** | | | | |
| **(B)** | | | | |
| **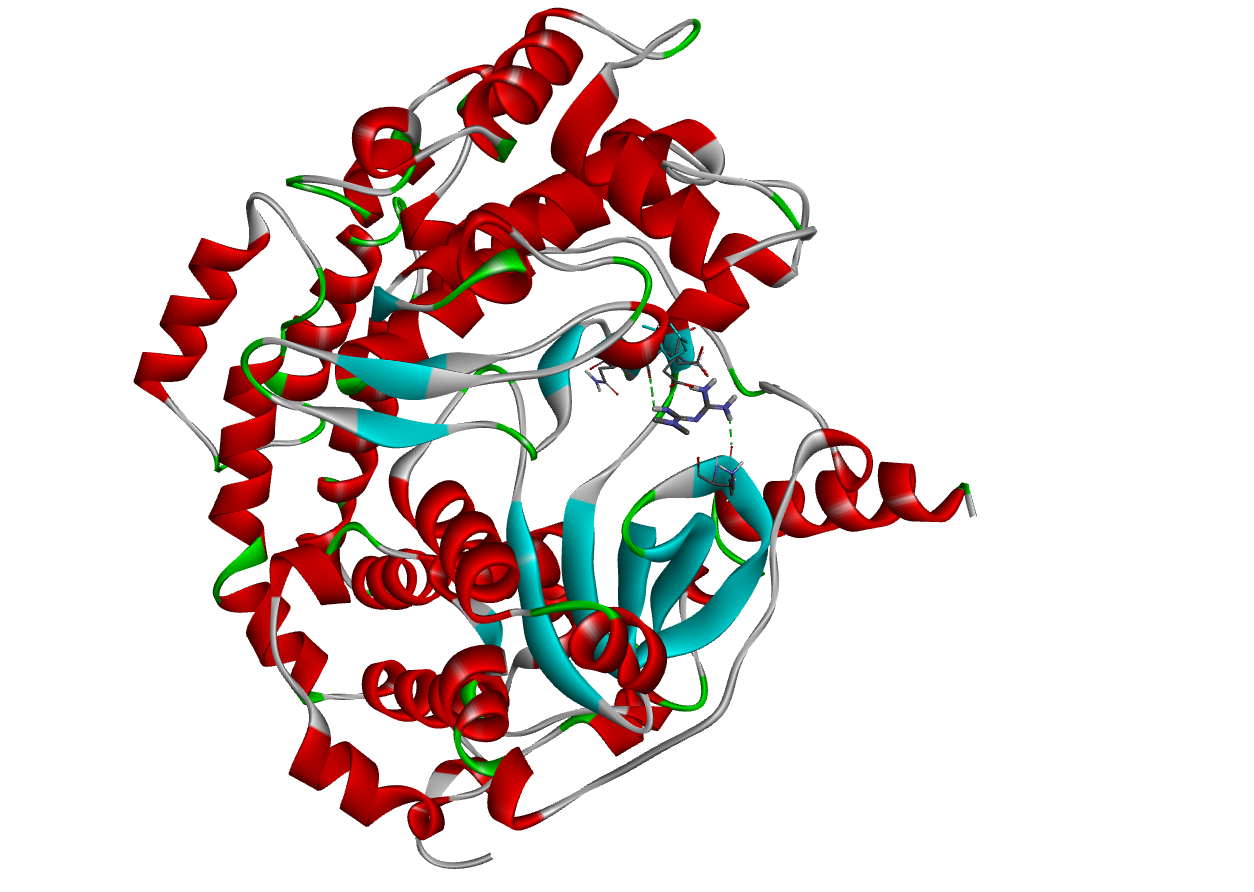** | | **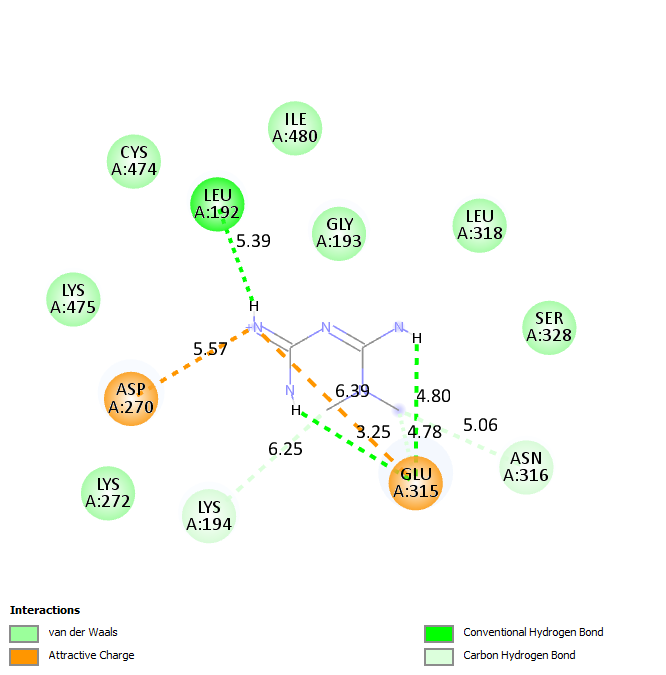** | | |
| **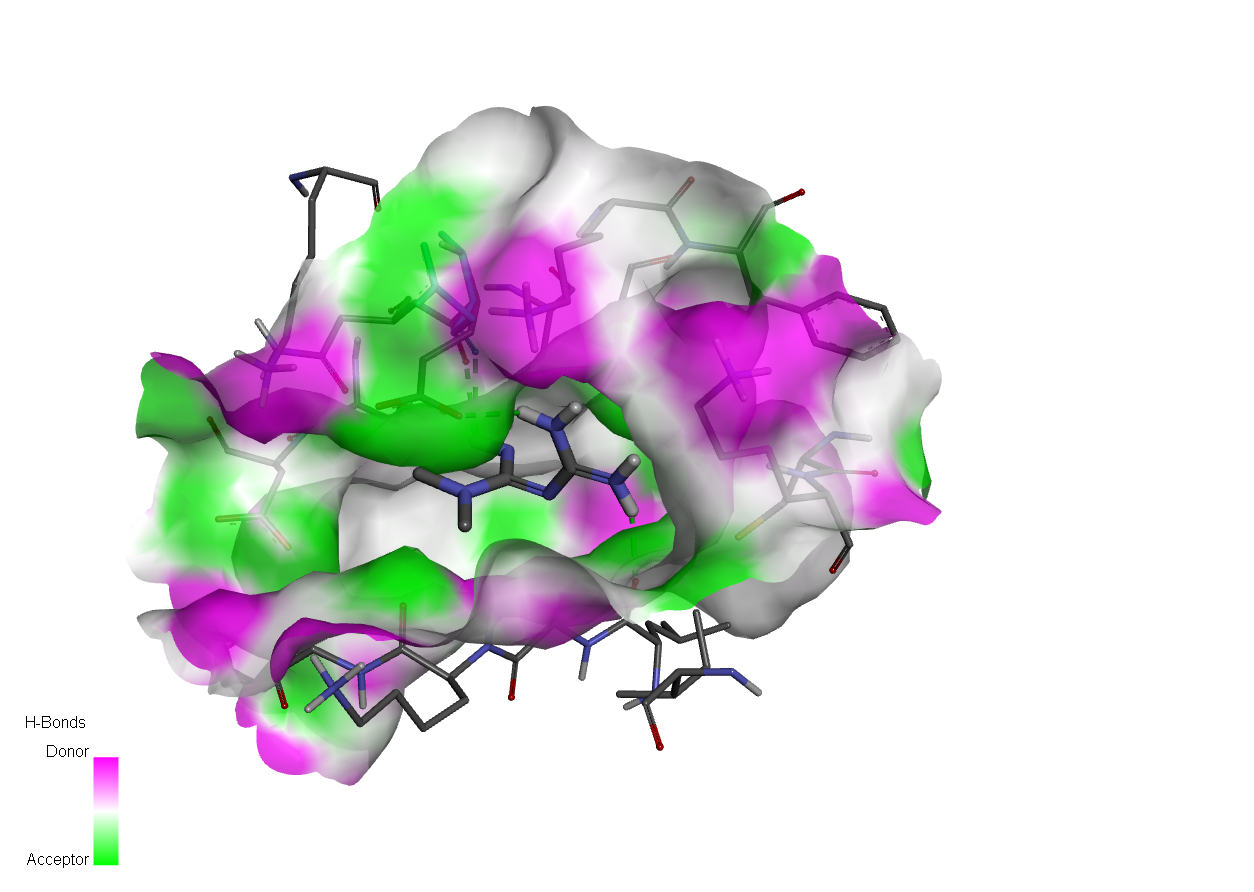** | | | | |
| **(C)** | | | | |

**Fig. 6 Docking analysis against GRK.** (**A**) Docking of BBR with GRK, (**B**) Docking of CAF with GRK and (**C**) Docking of MTF with GRK.


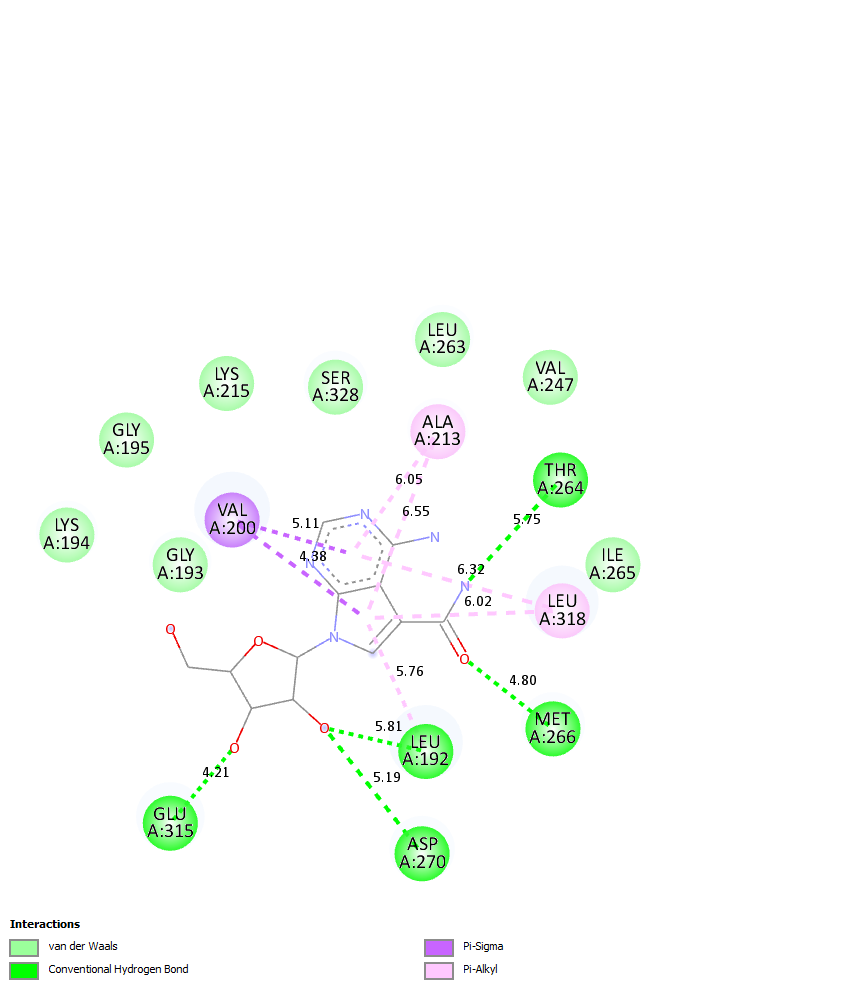


**Fig. 7 Docking of the co-crystal ligand with GRK.**
